# Global and regional changes in exposure to extreme heat and the relative contributions of climate and population change

**DOI:** 10.1038/srep43909

**Published:** 2017-03-07

**Authors:** Zhao Liu, Bruce Anderson, Kai Yan, Weihua Dong, Hua Liao, Peijun Shi

**Affiliations:** 1 State Key Laboratory of Earth Surface Processes and Resource Ecology, Beijing Normal University, Beijing, 100875, China; 2 Department of Earth and Environment, Boston University, Boston, 02115, USA; 3 Academy of Disaster Reduction and Emergency Management, Beijing Normal University, Beijing, 100875, China; 4 School of Geography, State Key Laboratory of Remote Sensing Science, Beijing Normal University, Beijing, 100875, China

## Abstract

The frequency and intensity of extreme heat wave events have increased in the past several decades and are likely to continue to increase in the future under the influence of human-induced climate change. Exposure refers to people, property, systems, or other elements present in hazard zones that are thereby subject to potential losses. Exposure to extreme heat and changes therein are not just determined by climate changes but also population changes. Here we analyze output for three scenarios of greenhouse gas emissions and socio-economic growth to estimate future exposure change taking account of both climate and population factors. We find that for the higher emission scenario (RCP8.5-SSP3), the global exposure increases nearly 30-fold by 2100. The average exposure for Africa is over 118 times greater than it has been historically, while the exposure for Europe increases by only a factor of four. Importantly, in the absence of climate change, exposure is reduced by 75–95% globally and across all geographic regions, as compared with exposure under the high emission scenario. Under lower emission scenarios RCP4.5-SSP2 and RCP2.6-SSP1, the global exposure is reduced by 65% and 85% respectively, highlighting the efficacy of mitigation efforts in reducing exposure to extreme heat.

Over the past decade tens of thousands of people have died from heat waves all over the world, for instance, Europe in 2003, Australia in 2008, Russia in 2010 and China in 2013^1^,^2^,^3^,^4^,^5^. Although the mortality attributable to extreme heat events has actually decreased over the last century due to medical progress and economic development^6^,^7^,^8^, it is expected to increase in the future under the influence of human-induced climate change as heat wave events become more intense and more frequent with longer duration over most land areas in the 21st century^9^, leading to increased risk of heat-related morbidity and mortality^10^,^11^. It is important to note, however, that the changes in heat-related morbidity and mortality are not only a function of changes in physical hazards resulting from human-induced climate change but also are a function of changes in the number and vulnerability of individuals exposed to these hazards^12^.

Accordingly, many studies have focused on future heat wave risk assessment^13^,^14^,^15^,^16^, which attempts to estimate the probable heat-related mortality and morbidity of people that an area would experience^13^,^17^ as a function of the changing characteristics of a given hazard, the number of people exposed to that hazard and their sensitivity and adaptive capacity to that hazard^12^,^18^. However, this last characteristic, which represents a population’s vulnerability to a particular hazard, can vary significantly for various factors such as age, season and geographic region^19^. Although a number of indices have been proposed focusing on different aspects of a population’s vulnerability, there is no consistent standard to measure vulnerability due to its inherent complexity^20^,^21^,^22^. In addition, projections of future demographic and socioeconomic factors of vulnerability are difficult to obtain, generally of coarse resolution and of short duration. As a result, most prior research uses fixed population estimates when quantifying future risk to changing climates, including those related to heat-induced mortality^10^,^23^. Here we extend this analysis by including future changes in the magnitude and spatial patterns of both extreme heat events and the number of people impacted by these events. We recognize that such an analysis ignores both the spatially and temporally non-linear and non-uniform vulnerability of these populations to these events and as such we do not attempt to estimate changes in heat-related mortality and morbidity explicitly. Instead, following the lead of Jones *et al*.^24^, we seek to quantify population exposure to extreme heat waves (referred to throughout simply as ‘exposure’), which is an important first step towards estimating global and regional scale changes in the risk of heat wave mortality and morbidity.

In particular, the primary purpose of this paper is to assess the future change of exposure to heat waves at global and regional levels under different development pathways. In addition, we also discuss the impact of three different factors: climate change, population growth and the interaction between the two. Further we select China, India and Nigeria as three typical countries to represent the different development paths and discuss their exposure change and the impact factors’ contribution. Based on this analysis of exposure and the relative contribution of different factors, we aim to characterize the future increase of heat-related exposure and clarify the importance of climate change and population growth to the change.

## Methods

### Materials

To estimate changes in the hazard we use bias-corrected and downscaled projections^25^ of Coupled Model Intercomparison Project Phase 5 (CMIP5) climate models to characterize future climate change. CMIP5 projections are based on general circulation model (GCM) estimates of present and future climate such as temperature and precipitation in response to various increases in radiatively active atmospheric constituents^26^,^27^. These increases are represented by four Representative Concentration Pathways (RCPs) corresponding to the atmospheric radiative forcing up to the year 2100^28^. Each RCP represents a pathway based on simulated influences of land use and emissions of aerosols and greenhouse gases (GHG). The highest pathway is RCP8.5 and depicts a scenario with radiative forcing rising to 8.5 W/m^2^ by 2100, without applying any mitigation policy to GHG emissions. The lower scenarios, including RCP6.0, RCP4.5 and RCP2.6, do adopt some mitigation measures to control the GHG emission^28^,^29^.

Following the lead of previous climate change impact assessments^24^, in this paper we subsequently use a subset of these models for which high spatial and temporal resolution downscaled data are available. In particular, we archive the 0.5° × 0.5°, daily output from the Inter-Sectoral Impact Model Intercomparison Project^30^, which is available for five representative CMIP5 GCMs (see Supplementary Table S1). Expanding upon Dong *et al*.’s^31^ research, we employ annual total heat wave days (HWDs) to quantify the heat hazard. This metric is defined as the total days that daily maximum temperatures exceed a given region’s threshold for at least three consecutive days, where the threshold here is defined as the 95th percentile value for daily maximum temperatures over the historical reference period (1971–2000)^32^. If the local 95th percentile is lower than 25 °C, we set 25 °C as the threshold^33^. Here we chose to use a relative threshold instead of a fixed threshold because we are interested in estimating changes in exposure across broad expanses of the globe, for which no single fixed threshold suffices^34^. Instead, we chose the 95th percentile threshold as the simplest way to define regionally relevant heat waves across the globe. We note that results throughout are robust to the use alternate heat wave durations (e.g. at least six consecutive days) and different thresholds (e.g. the 90th or 97.5th percentile value for daily maximum temperatures) and different heat-related hazard metrics, including accumulated heat (AH), which refers to the accumulated temperature exceedances above the threshold temperature during heat wave events (details can be found in Supplementary Discussion 1; results can be found in Supplementary Figures).

To estimate changes in population, we use projections from the Shared Socioeconomic Pathways (SSPs). SSP scenarios describe alternate evolutions of the socio-economic system and the eco-environment^35^ and employ various mitigation and adaptation strategies in response to socioeconomic challenges^36^. There are five SSP scenarios namely SSP 1–5. SSP1, SSP2, and SSP3 denote low, intermediate and high challenges, respectively; SSP4 and SSP5 are dominated by adaptation challenges and mitigation challenges, respectively^35^. The SSP scenario data provide gridded population data at 0.5° × 0.5° spatial resolution and yearly temporal resolution from 1950 to 2100 and can be downloaded from the ISI-MIP website.

Based upon the four RCP scenarios and five SSP scenarios, we can generate a 4 × 5 climate/population matrix^37^ although some RCP-SSP combinations (e.g., RCP2.6-SSP3) are unlikely to arise in practice. These combinations provide the basis for future heat-related exposure here and three combinations—namely, RCP2.6-SSP1, RCP4.5-SSP2, and RCP8.5-SSP3—have been selected for further analysis.

### Methods

For both the base period (1971–2000) and the future period (2071–2100), this paper uses 30-year averages of HWDs and population in order to minimize inter-annual variations in each. Exposure is defined here as population exposed to heat waves, and is computed at each grid point by multiplying annual total number of HWDs and population together for both base and future periods^24^. Thus, the unit of exposure is person-day. For each of the five climate model projections we calculate 30-year mean exposure over the base period and future period. In addition, we aggregate grid cells’ exposure to global, continental and country scale. As noted above, our interest here is to determine changes in exposure to regional heat waves, not necessarily the changing risk of mortality/morbidity to these heat waves, which would require inclusion of vulnerability estimates. We recognize that different countries may experience different risks given the same change in exposure due to differences in the sensitivity and adaptability of the affected populations; however estimating the changes in vulnerability, and by extension risk, is outside the scope of the research conducted here.

Instead, we adopt the method from Jones *et al*.^24^ to assess the influence of climate and population on exposure. We decompose the change of exposure into three effects and calculate each effect’s relative importance using equation (1). Specifically, three types of effects are measured:

- The climate effect. We measure the influence of climate on exposure by allowing climate to change according to the model projection but leaving population fixed at the base period level.
- The population effect. Similarly, we measure the population effect by allowing population to change but leave the climate fixed at the base period level.
- The interaction effect. It is defined as the total exposure change minus the summation of climate and population effect changes and is the result of simultaneous variations in both climate and population.

The decomposition equation for exposure change used in this paper is equation (1).


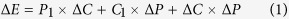


In this equation, Δ*E* is the total change in exposure. *C*_1_ and *P*_1_ are the HWD and population in 1971–2000, and Δ*C* and Δ*P* are the change in HWD and population from 1971–2000 to 2071–2100. Here we will refer to *C*_1_ × Δ*P* as the population effect, *P*_1_ × Δ*C* as the climate effect and Δ*C* × Δ*P* as the interaction effect.

If we divide both sides of the equation (1) with *E*_1_, the equation provides an estimate of the percentage change for each indicator (equation 2).


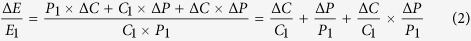


where 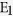 is the exposure in 1971–2000 such that 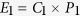. 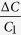 and 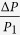 are the percentage change for climate and population from 1971–2000 to 2071–2100. Equation (2) will be used to discuss the quantitative relationships between the interaction effect, climate effect and population effect.

## Results

### Spatial distribution pattern and statistics of global and continental exposure

Overall, global aggregate annual exposure for the 1971–2000 period is 57 billion person-days on average (Supplementary Fig. S1). In comparison, the exposure increases by ~18–37 times to ~1200–2300 billion person-days (Supplementary Fig. S2) by the end of 21 century under the RCP8.5-SSP3 scenario. On average, exposure increases by 27, 10 and 4 times for the RCP8.5-SSP3, RCP4.5-SSP2 and RCP2.6-SSP1 scenarios respectively. Since results are qualitatively consistent across all models, we present the average values of the five models in the following figures. Results from lower emission scenarios and individual models can be found in the Supplementary Figs S3 and S4.

**Figure 1:**
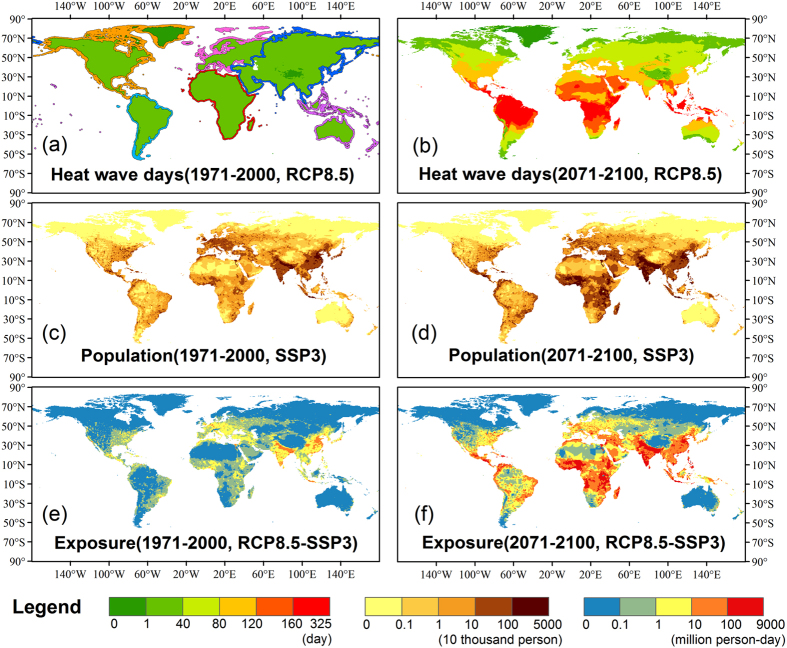
Multi-model average of heat wave days (**a**,**b**; left legend), population (**c**,**d**; middle legend), and exposure (**e**,**f**; right legend) averaged for the period 1971–2000 (left: **a**,**c**,**e**) and the 2071–2100 period (right: **b**,**d**,**f**) under scenario RCP8.5-SSP3. The six continental regions analyzed below designated by the colored boundaries in (**a**). These maps were generated using ArcMap 10.3, visit http://desktop.arcgis.com/en/ for more details.

To examine the global change in exposure further, Fig. 1 shows the spatial patterns of global exposure and its components for the 1971–2000 and 2071–2100 periods under RCP8.5-SSP3. Generally, globally-averaged HWDs during the 1971–2000 period are under 15 days per year (Fig. 1a), whereas it increases substantially during the 2071–2100 period. In certain low latitude areas, it is expected that annual HWDs will increase by over 150 days during the future period. In contrast, HWDs increase by 80–120 days in the mid-latitudes and 40–80 days in the sub polar regions. With regard to changes in population (Fig. 1c,d), it is projected that population will grow very rapidly in sub-Saharan Africa, Central America and South Asia. In contrast, for most of North America, Europe, and northern Asia (including China), the population change is small and in some cases negative.

Figure 1e,f show the resulting spatial patterns of global exposure for the 1971–2000 and 2071–2100 periods under RCP8.5-SSP3. During the historical period (1971–2000), geographic variations in exposure are generally a function of population (given the relatively constant HWD values) and tend to be highest in the Indian subcontinent and parts of China. By the 2071–2100 period, exposure remains high in these regions, however there are now substantial portions of the globe that are expected to have similar exposure rates, particularly across much of the tropical and sub-tropical regions. Indeed, during this period it is expected that exposure will be highest in Nigeria, parts of Central America, and Indonesia, for instance. However even in higher latitude regions such as North America and Western Europe, exposure is expected to increase, principally as a result of increased numbers of HWDs (given that the change in population is near-zero – see below).

To better discern the magnitude of these exposure values and their changes over time, we aggregate the values across six continental-scale regions (Supplementary Fig. S5). As noted before, initial exposure values are highest in Asia. However, by the period 2071–2100, these are matched or even exceeded by those in Africa. In turn, Africa experiences the greatest increase in exposure from 6 billion person-days to ~700 billion person-days, equivalent to nearly 120 times its current value. Asia also experiences a substantial increase as well from 36 billion person-days to ~600 billion person-days, equivalent to an absolute growth of ~570 billion. In contrast, Europe, North America, and South America all experience much smaller absolute changes in exposure. Causes for these continental differences are discussed below.

### Global, continental, and country-scale comparison of exposure effects

**Figure 2:**
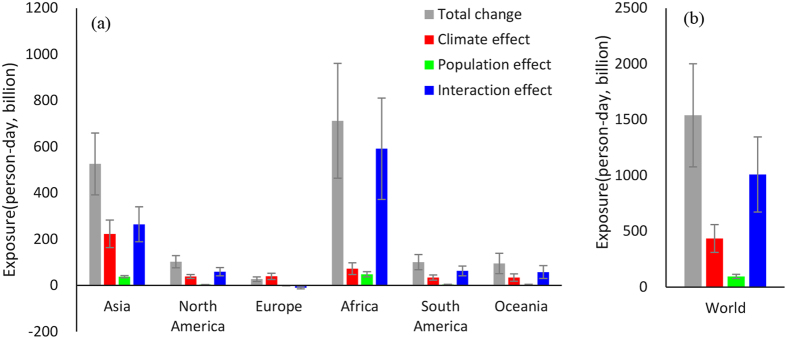
Decomposition of aggregate regional (**a**) and global (**b**) projected change in exposure under RCP8.5-SSP3 scenario. Multi-model average increase in total projected exposure change (gray), exposure change from the climate effect (red) keeping population constant; exposure change from the population effect (green) keeping climate constant; and exposure change from the interaction effect (blue) between climate and population – see Methods Section for details. Error bars illustrate the standard deviation in total projected exposure change across the models for each region/effect.

To discern the drivers for the relatively large (and small) increases in exposure over various continental-scale regions, Fig. 2 shows the change in exposure and its components for different regions (and the globe as a whole) under the RCP8.5-SSP3 scenario (individual model results are shown in Supplementary Fig. S6 for global aggregation and Supplementary Fig. S7 for continental aggregations). At the global level, the change of exposure caused by the climate effect is approximately 28% of the total, whereas the change of exposure caused by the population effect is only ~6%; in contrast the change in exposure due to the interaction effect comprises ~66% of the total. These results highlight the importance of the synergistic interactions between changing populations and climate in driving enhanced exposure under the high emission scenario. Under RCP4.5-SSP2, the interaction effect comprises ~52% of total exposure change, and remains the most prominent contributor to overall change in exposure. However, under the RCP2.6-SSP1 the interaction effect contribution decreases to ~36% for RCP2.6-SSP1, while the climate effect increases to ~49% (Supplementary Fig. S8). Importantly, under all three scenario combinations, the climate effect is always substantially larger than the population effect. Indeed, absent any change in climate the global aggregate exposure would be reduced by 85%, 90% and 94% for the RCP2.6-SSP1, RCP4.5-SSP2 and RCP8.5-SSP3 scenarios respectively, which highlights the relatively minor role future changes in population (on their own) play in enhancing future rates of extreme heat exposure.

Turning to the continental level (Fig. 2a) we find significant differences in the relative importance among climate, population, and interaction effects across regions. As noted earlier the largest increase in total exposure is expected to occur in Africa, followed by Asia. However, the predominant drivers of these increases differ between the two regions. In Africa, the largest driver is the interaction effect resulting from the combined increases in both HWDs and population. In contrast, in Asia (as well as in Oceania, North and South America), the interaction effect is still the largest driver overall however there is also a substantial contribution from the climate effect as well, highlighting that even in the absence of population increases changing temperatures by themselves would amplify the exposure rates in these regions. In Europe, where the overall population is expected to decrease, both the population and interaction effect are negative such that the overall change in exposure is less than expected when considering changes in climate on its own. Finally, as with the globally-aggregated results, the population effect, which considers the change in exposure absent any change in climate, contributes less than 7% to the total change in exposure across all 6 regions, again highlighting the relatively minor role future changes in population (on their own) play in enhancing future rates of exposure to extreme heat. We also did the same analysis for the other two scenarios (Supplementary Fig. S8). With regard to the change in total exposure, we find that alternate emissions scenarios reduce the global aggregate exposure relative to scenario RCP8.5-SSP3 by 86% and 64% under scenario RCP2.0-SSP1 and scenario RCP4.5-SSP2 respectively, with similar sized reductions across all six continental-scale regions. Further, given the relatively small change in the population effect across the various SSPs, we find that these reductions are driven almost entirely by changes in the climate and interactions effects, both at the globally-aggregated scale as well as the continental scale.

**Figure 3:**
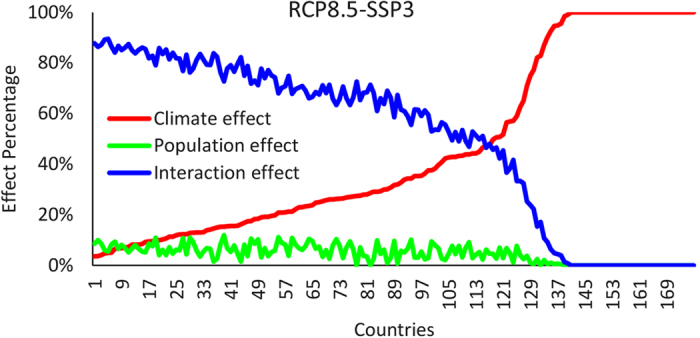
Fractional contribution to aggregate country-wide projected change in exposure under RCP8.5-SSP3 from the climate effect (red), population effect (green) and interaction effect (blue). For clarity countries sorted by the fractional contribution of the climate effect. For countries with negative population growth, climate effect is set to 100%.

To further refine the geographic structure of exposure, we next decompose every country’s average exposure change under the RCP8.5-SSP3 into the contribution from the climate effect, population effect and interaction effect, retaining results for the largest 175 countries (Fig. 3). For most countries (66%) the interaction effect is the main contributor while the climate effect is the main contributor for the remaining 34% of the countries. In no country is the population effect the main contributor, at least under the RCP8.5-SSP3 (for the RCP2.6-SSP1 and RCP4.5-SSP2 the percentage contribution of the population effect on the overall change does tend to increase, although it consistently remains below the contribution from the interaction effect - Supplementary Fig. S9). Indeed, the population effect consistently contributes only about 10% or less to the total exposure change.

**Figure 4:**
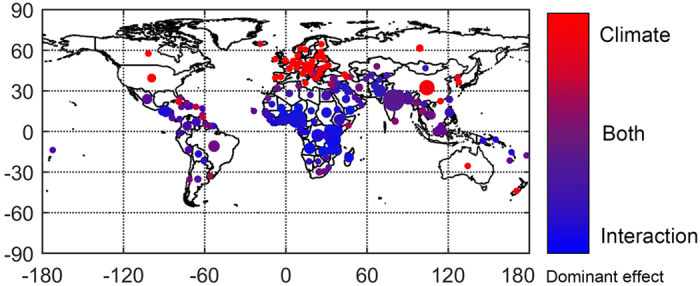
Decomposition of aggregate country level projected change in exposure for RCP8.5-SSP3 scenario. The size of the circle refers to total change. Red shading is proportional to the fractional contribution from the climate effect and blue shading is proportional to the fractional contribution from the interaction effect. The fractional contribution from the population effect is also shown and is represented by green shading but for clarity green is not included on the color bar because the effect is small everywhere. This map was generated using Matlab 2015a, visit http://www.mathworks.com/ for more details.

To discern the geographic structure of each effect’s contribution to the total change in exposure Fig. 4 shows the total exposure change for each country, as well as the weighted contribution by the climate effect compared to the interaction effect (the population effect is also included, however because of its relatively small influence discussed above, it is not represented on the color bar). In general, mid- to high-latitude countries tend to be influenced predominantly by the climate effect, principally because the population changes in these countries tend to be relatively small or even negative; importantly, this holds true for China as well. At lower latitudes, the overall change in exposure tends to either result predominantly from the interaction effect (which is the primary driver across much of Africa) or from the combination of the climate and interaction effects (which tends to be the case for Southeast Asia including India, as well as Central and South America). To explore the importance of various drivers of exposure change at the country level, below we select three representative countries—namely China, India, and Nigeria—for further analysis.

### Drivers of exposure change in China, India and Nigeria

Based upon the magnitude and characteristics of the overall exposure change discussed above, we select China (predominantly climate influenced), Nigeria (predominantly interaction influenced) and India (jointly influenced by climate and interaction effects) as representative countries for further analysis. Overall, China’s and India’s initial exposure are fairly similar and are more than ten times that of Nigeria (Supplementary Figs S10 and 11). By the end of the century, under the RCP8.5-SSP3 scenario, India’s exposure increases by a factor of 17 and is nearly twice as large as that of China (which increases by only a factor of 7). Interestingly, by the end of the century, Nigeria’s exposure, which increases by a factor of nearly 130, is equivalent to, and possibly exceeds, that of China. In contrast, for the RCP2.6-SSP1 scenario, the growth of exposure for the three countries is much less, resulting in a 66–87% reduction in compared to the RCP8.5-SSP3 scenario.

**Figure 5:**
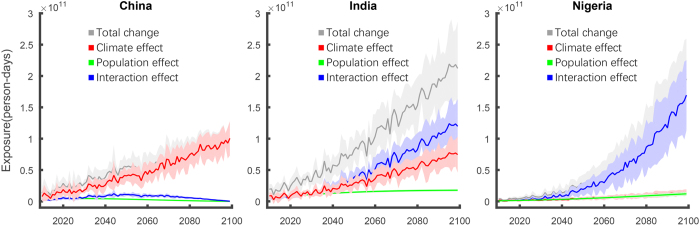
Decomposition of projected annual aggregate country-level change in exposure for China, India, and Nigeria under RCP8.5-SSP3 scenario. Multi-model average increase in total projected exposure change (gray), exposure change from the climate effect (red) keeping population constant; exposure change from the population effect (green) keeping climate constant; and exposure change from the interaction effect (blue) between climate and population – see Methods Section for details. Shadows illustrate the standard deviation in total projected exposure change across the models for each country/effect.

To analyze the temporal trend of exposure change and the contribution of each effect, we calculate the annual exposure from 2010 to 2100 (Fig. 5). As noted above, the main contributors to the overall exposure in the three countries differs consistently over time. Under RCP8.5-SSP3, the exposure change of China is mainly affected by climate, while the population effect and interaction effect are small but positive; the climate effect becomes even more prominent under RCP4.5-SSP2 and RCP2.6-SSP1, in which China’s population decreases after 2030 s such that the population and interaction effect are negative and the overall exposure is less than expected when only accounting for changes in climate (Supplementary Figs S13 and S14). In contrast, in Nigeria, the overall exposure change is dominated by the interaction effect under RCP8.5-SSP3, despite relatively small contributions from changes in climate and population alone, which indicates that the synergistic combination of both rising temperatures and very rapid population increases are giving rise to enhanced exposure. India’s exposure change under the RCP8.5-SSP3 is mainly influenced by the interaction effect however there is also a substantial contribution from the climate effect; in combination the overall change subsequently exceeds that of both Nigeria and China and is the largest of any country analyzed here (see Fig. 4). In this country, then, it is the change in climate acting on both the large base population (the climate effect) and the large change in population (the interaction effect) that produces the substantial increases in exposure. It is also important to note that with the exception of Nigeria in the low-emission scenario (Supplementary Figs S13 and S14), the population effect is the smallest contributor of the three, which indicates that absent a change in climate the change in exposure in these three countries would be substantially reduced. Indeed, Fig. 3 suggests the reduction would be on the order of 85–100% for all countries analyzed here.

Finally, we note here that in most countries, the overall change in exposure to future extreme heat is mainly (as in Nigeria) or partly (as in India) caused by the interaction of global warming and population growth (see Figs 3 and 4). Equation (2) helps explain why the interaction effect can be so substantial in many countries and continents. Indeed, if 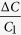 and 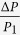 are both larger than 1 then the interaction effect can become more important than either of the two individual effects. Equivalently, both HWD and population must increase by at least 100% from 1971–2000 to 2071–2100 such that the third term of Equation (2) 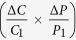 is larger than 1. To show the importance of this interaction on a continental scale, we decompose the projected percentage change in exposure under RCP8.5-SSP3, and aggregate the values across 6 continental-scale regions (Supplementary Fig. S15). For Africa, which has the largest overall percentage increase in exposure as well as the largest percentage increase induced by the interaction effect, HWDs increase by over 1200% (from ~10 days per year to ~140 days per year, equivalent to a factor of 12) while population increases by 800% on average (from 0.5 billion to 4.7 billion) such that the change induced by their interaction is approximately 10000% (equivalent to a factor of 100). Similarly, the percent change in exposure induced by the interaction effect in South America is expected to be 5000% (equivalent to a factor of 50). In contrast, for regions with stable (North America) or negative (Europe) population growth estimates, the interaction term (along with the population term) is relatively small compared to the percentage change induced by the changing climate alone.

## Discussion

Here we have analyzed the changing exposure of the world’s populations to extreme heat waves using global climate models’ and social economic models’ projection for both changes in climate and population under high and low emissions/growth scenarios. Overall, the global total exposure is expected to increase by approximately a factor of 30 from 1971–2000 to 2071–2100 under the high emissions (RCP8.5) and population growth (SSP3) scenario. For certain regions, such as much of Africa, the exposure could increase by over a factor of 120, while for others, such as the mid- to high-latitude regions of the Northern Hemisphere, the increase is less than a factor of 5.

For most regions and countries in the world (including much of Africa, the Middle East, South and Central America, Southeast Asia, North Oceania islands and India) the change in exposure to extreme heat is mainly caused by the synergistic interaction between rapidly increasing populations and large increases in the number of heat-wave days during the year. In other regions of the world where population growth is expected to be low or negative —again principally confined to the mid- to high-latitude regions of the Northern Hemisphere but also China—increases in exposure result primarily from increases in temperature. Importantly, in no region/country is the change in exposure predominantly the result of the changing population alone; indeed across all countries, the change in population by itself only contributes 0–15% to the overall change in exposure. Because of the secondary role of changing populations upon overall exposure, expected changes in exposure are more sensitive to future changes in concentrations of greenhouse gases such as carbon dioxide than they are to future changes in population growth such that under lower emission scenarios— RCP4.5-SSP2 and RCP2.6-SSP1 respectively—future exposure can be reduced by 66–87% relative to that under high emissions scenarios. This result reconfirms the benefits of climate mitigation efforts^38^ for reducing overall exposure to future heat extremes.

It is important to note that while in this study we define heat waves as periods in which the 95th percentile threshold temperature is exceeded for a duration of at least 3 days (95P3D), we also analyzed the results with other thresholds/threshold (e.g. 90% and 6 days’ duration – 90P6D; 97.5% and 3 days’ duration – 97.5P3D). Overall, the patterns of exposure change and their drivers based on these three definitions are qualitatively and quantitatively similar (Supplementary Figs S16–S25). In addition, an analysis of alternate exposure metrics—in particular accumulated excessive temperatures during heat waves—indicates that the patterns of exposure change and their drivers are similar at all levels of aggregation (global, continental and country— Supplementary Figs S18–23). While it recognized that the absolute values of exposure and exposure change will depend upon both the heat metric and threshold temperature used, these sensitivity studies highlight the robustness of our overall results.

At the same time, as noted in the introduction, in this work we only analyzed the change in exposure to extreme heat as a function of a change in the hazard (number of heat wave days or accumulated heat) and population. To properly estimate a change in risk of mortality/morbidity resulting from this exposure, demographic and socioeconomic characteristics such as age, gender, per capita income and education level should be included into the analysis^16^,^39^,^40^,^41^,^42^. However, since projections of these characteristics tend to be relatively coarse and of low confidence, we have not included the demographic and socioeconomic factors in our analysis.

Further, it is recognized that in some locations such as the tropics threshold-based metrics can produce relatively large exceedance estimates depending upon how the magnitude of daily temperature variations—which tend to be smaller than in the mid-latitudes—compares with the expected overall increase in temperatures, which also tend to be smaller; further temperatures in these regions are already relatively high and hence closer to absolute human-tolerance levels. For these reasons, a fixed, absolute threshold may be more appropriate for these regions. However, as noted for this study we chose to use a relative threshold instead because we are interested in estimating changes in exposure in a consistent way across broad expanses of the globe, for which no single fixed threshold suffices^34^.

Another limitation of this study is the use of high-resolution downscaled climate data from a limited number of CMIP5 GCMs using a single downscaling methodology. While the use of a subset of high-resolution climate model output is consistent with many climate impact and assessment studies—including the paper of Jones *et al*.^24^, which used downscaled data (at the same resolution) from 4 GCMs—we recognize that five models cannot fully represent the range of outcomes (potentially) present in the full suite of CMIP5 GCMs, although as noted we find the relative change in exposure and the importance of various drivers of that change are the same across all five models, suggesting the overall results presented in the paper are robust to the inclusion of a larger suite of models. Further, we recognize that the statistical downscaling methods used here, while computationally efficient, preserve many of the large-scale characteristics of the GCMs and as such may not provide higher-resolution dynamic and thermodynamic refinement of these characteristics as found, e.g. in more computationly intensive numerical downscaling methods. This set of caveats is well recognized^30^ and is applicable to the study of any complex system—whether biological^43^, physical^44^, or socio-economic^45^ —in which a hierarchy of models is needed to advance our understanding of the important drivers, responses, and uncertainties of the system. At the same time, this recognition highlights the need for a method to conextualize a given study within that hierarchy based upon a rubric of modeling characteristics that places the study along a spectrum of model complexity so future research can refine the results (e.g. by moving up the hierarchy), or theorize the results (e.g. by moving down the hierarchy). Further, minimum thresholds could be set for studies within a given subset of the model hierarchy; climate model data could be designated by their placement along the spectrum; and users of climate model data could determine the level of complexity most appropriate for their studies.

To conclude, we feel the consideration of changing demographics, inclusion of more downscaled CMIP5 data and application of climate-specific thresholds to be worth analyzing in the future.

## Additional Information

**How to cite this article:** Liu, Z. *et al*. Global and regional changes in exposure to extreme heat and the relative contributions of climate and population change. *Sci. Rep.* **7**, 43909; doi: 10.1038/srep43909 (2017).

**Publisher's note:** Springer Nature remains neutral with regard to jurisdictional claims in published maps and institutional affiliations.

## Supplementary Material

Supplementary Information
