# Supplementary material for: Global and regional changes in exposure to extreme heat and the relative contributions of climate and population change
Source: Sci Rep. 2017 Mar 7;7:43909. doi: 10.1038/srep43909 (PMC5339717; doi:10.1038/srep43909)
Supplement: Supplementary Information [file srep43909-s1.pdf]

Global and regional changes in exposure to extreme heat and the relative contributions of climate and population change

Zhao Liu[1,2,3,*], Bruce Anderson[2], Kai Yan[4], Weihua Dong[4,*], Hua Liao[4] and Peijun Shi[1,3,*]

[1] State Key Laboratory of Earth Surface Processes and Resource Ecology, Beijing Normal University, Beijing, 100875, China.

[2] Department of Earth and Environment, Boston University, Boston, 02115, USA.

[3] Academy of Disaster Reduction and Emergency Management, Beijing Normal University, Beijing, 100875, China.

[4] School of Geography, State Key Laboratory of Remote Sensing Science, Beijing Normal University, Beijing, 100875, China.

Correspondence and requests for materials should be addressed to P.S. (email: spj@bnu.edu.cn) or W.D. (email: dongweihua@bnu.edu.cn)

Supplementary Discussion 1. The Accumulated heat definition

Here accumulated heat (AH) refers to the total temperatures that exceeded the threshold temperature in heat wave events. This indicator combines heat wave days and anomaly temperature together. The definition of heat wave day is same and we choose 95th percentile as the threshold. Every heat wave day's exceeded temperature is added together (shown as formula 1). Exposure is defined here as population exposed to heat, and is computed at each grid point by multiplying annual total ℃ of AH and population together for both base and future periods. Thus, the unit of exposure is person- ℃. Since many researchers have studied the percentage change in mortality per 1 ℃ temperature increase [1], this accumulated heat indicator can be easily use to estimate heat-related mortality change.

$$\text{Accumulated Heat} = \sum_{i=1}^{HWD}(Temp_{max}(i) - Temp_{Threshold}) \qquad (1)$$

1 Hajat, S., & Kosatky, T. (2010). Heat-related mortality: a review and exploration of heterogeneity. *Journal of Epidemiology and Community Health*, **64**(9), 753–760.

Supplementary Table S1. Overview of the GCMs

Name	Institute	References
GFDL-ESM2M	Geophysical Fluid Dynamics Laboratory	(Dunne et al. 2012); (Dunne et al. 2013)
HadGEM2-ES	Met Office Hadley Centre	(Jones et al. 2011)
IPSL-CM5A-LR	Institute Pierre-Simon Laplace	(Mignot and Bony 2013)
MIROC-ESM-CHEM	Japan Agency for Marine-Earth Science and Technology, Atmosphere and Ocean Research Institute (the University of Tokyo), and National Institute for Environmental Studies	(Watanabe et al. 2011)
NorESM1-M	Norwegian Climate Centre	(Bentsen et al. 2012); (Iversen et al. 2012)

Reference:

Bentsen M et al. (2012) The Norwegian earth system model, NorESM1-M-Part 1: Description and basic evaluation Geoscientific Model Development Discussions 5:2843-2931. doi:10.5194/gmdd-5-2843-2012

Dunne JP et al. (2012) GFDL's ESM2 global coupled climate-carbon Earth System Models. Part I: Physical formulation and baseline simulation characteristics Journal of Climate 25:6646-6665. doi:10.1175/jcli-d-11-00560.1

Dunne JP et al. (2013) GFDL’s ESM2 Global Coupled Climate–Carbon Earth System Models. Part II: Carbon System Formulation and Baseline Simulation Characteristics* Journal of Climate 26:2247-2267. doi:10.1175/jcli-d-12-00150.1

Iversen T et al. (2012) The Norwegian earth system model, NorESM1-M–Part 2: Climate response and scenario projections Geosci Model Dev Discuss 5:2933-2998. doi:gmdd-5-2933-2012

Jones C et al. (2011) The HadGEM2-ES implementation of CMIP5 centennial simulations Geoscientific Model Development 4:543-570. doi:10.5194/gmd-4-543-2011

Mignot J, Bony S (2013) Presentation and analysis of the IPSL and CNRM climate models used in CMIP5 Climate Dynamics 40:2089-2089. doi:10.1007/s00382-013-1720-1

Watanabe S et al. (2011) MIROC-ESM 2010: model description and basic results of CMIP5-20c3m experiments Geosci Model Dev 4:845-872. doi:10.5194/gmd-4-845-2011

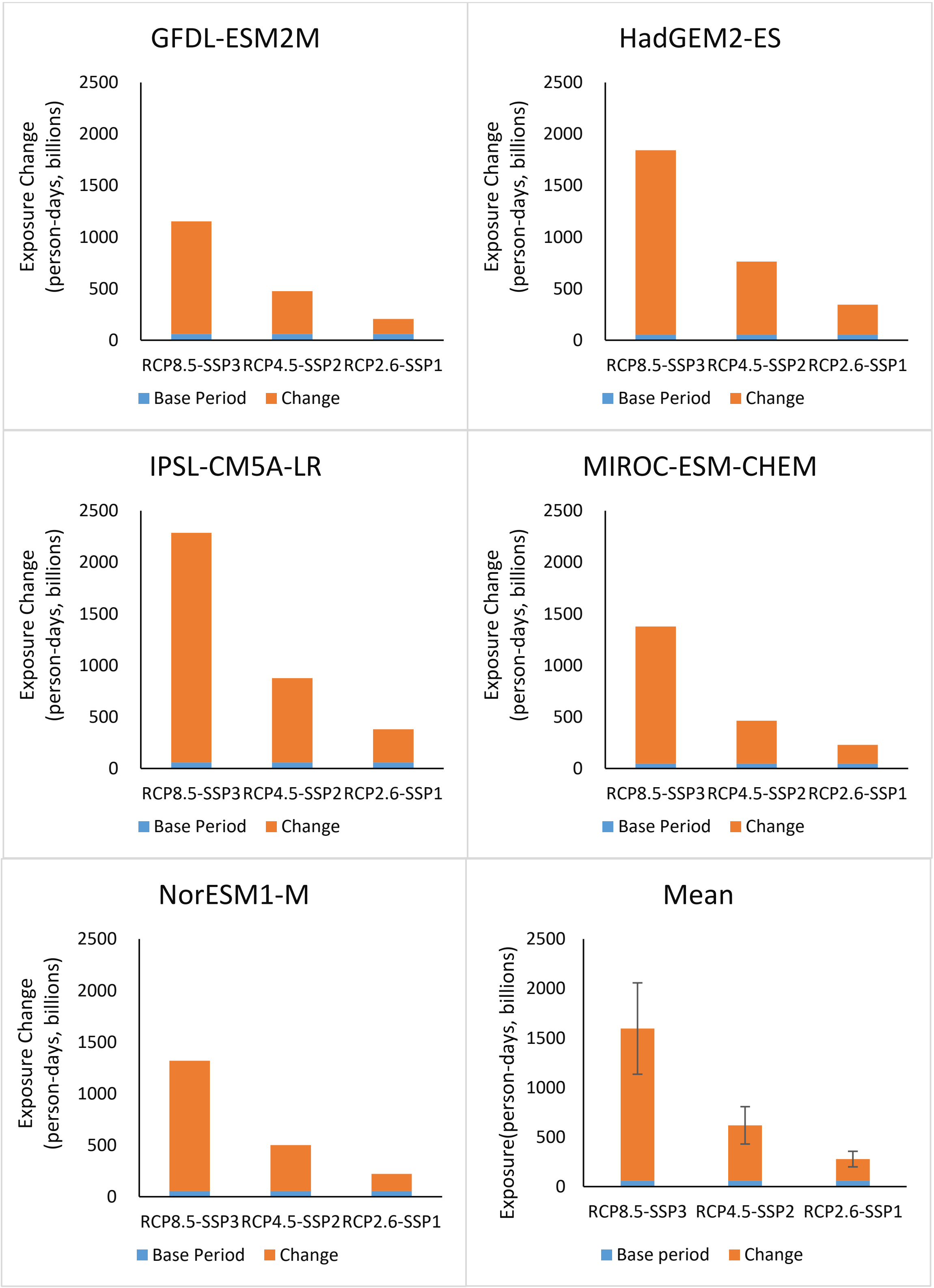

Supplementary Figure S1 Five models and multi-model average of global aggregate exposure in the base period (blue) and projected change (orange) under three scenarios. Error bars illustrate the standard deviation in projected exposure change across the models.

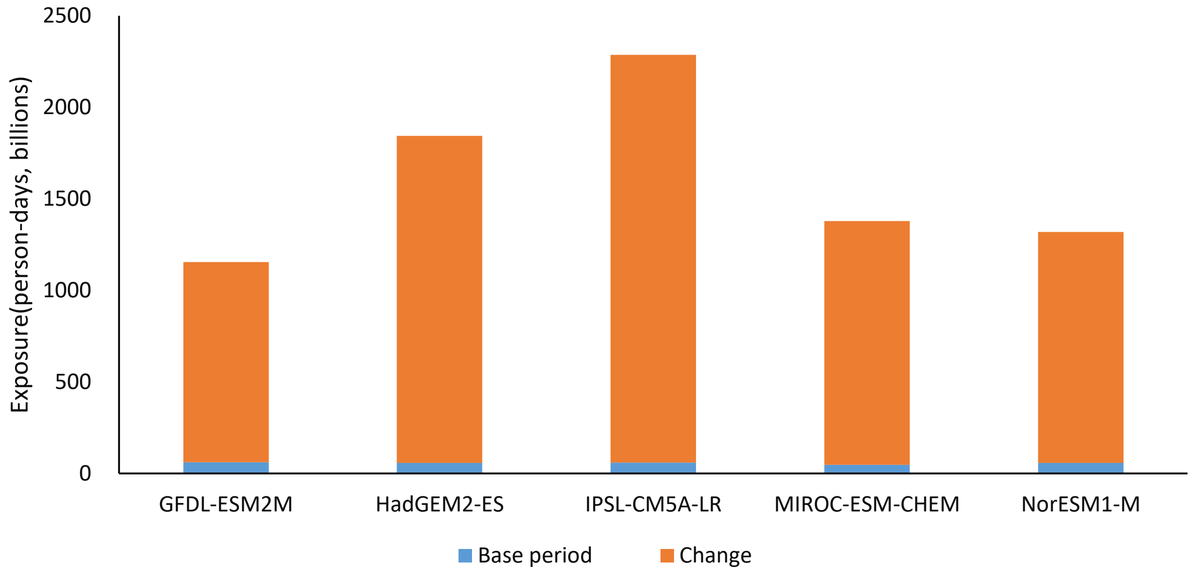

Supplementary Figure S2 Global aggregate exposure in the base period (blue) and projected change (orange) under RCP8.5-SSP3 scenario for five models used in this study.

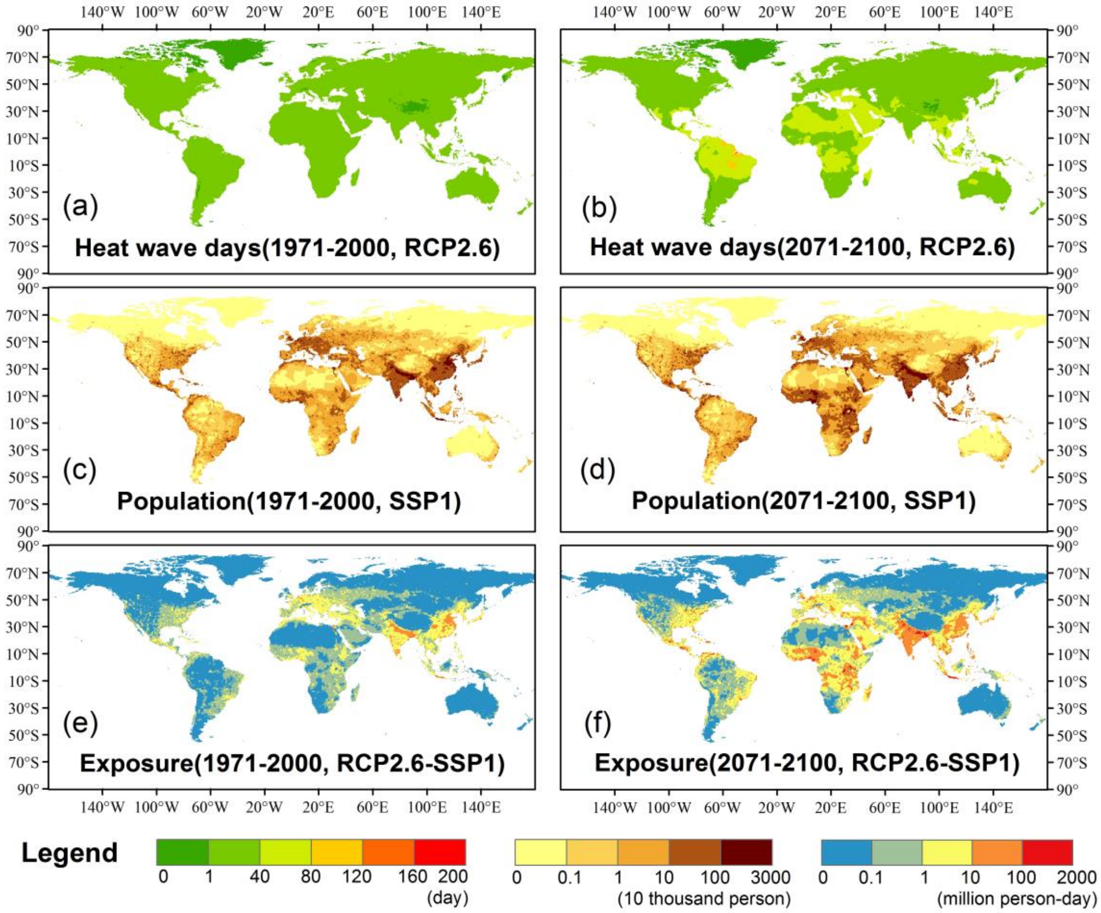

(a)
Heat wave days(1971-2000, RCP2.6)
(b)
Heat wave days(2071-2100, RCP2.6)
(c)
Population(1971-2000, SSP1)
(d)
Population(2071-2100, SSP1)
(e)
Exposure(1971-2000, RCP2.6-SSP1)
(f)
Exposure(2071-2100, RCP2.6-SSP1)
Legend
0 1 40 80 120 160 200
(day)
0 0.1 1 10 100 3000
(10 thousand person)
0 0.1 1 10 100 2000
(million person-day)

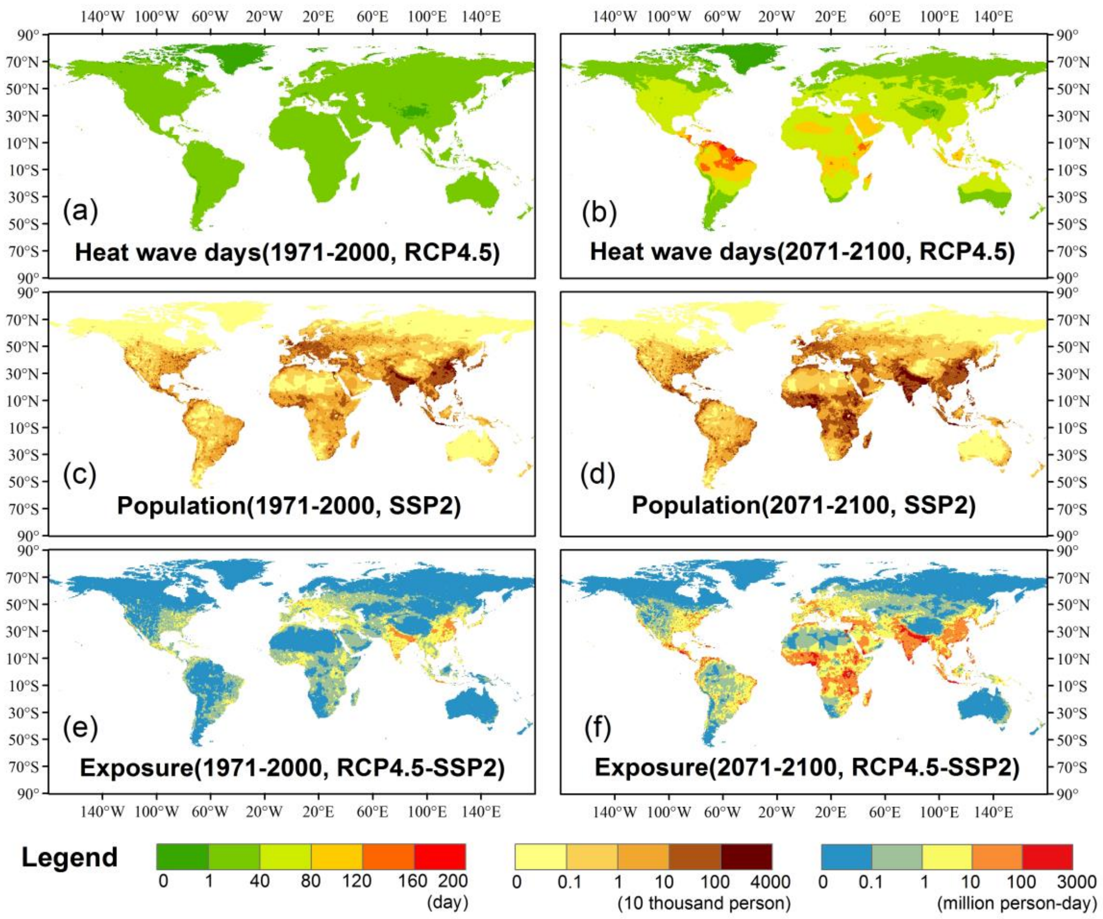

Supplementary Figure S3 Multi-model average of heat wave days (a, b; left legend), population (c, d; middle legend), and exposure (e, f; right legend) averaged for the period 1971-2000 (left: a, c, e) and the 2071-2100 period (right: b, d, f) under scenario RCP2.6-SSP1 (first panel) and RCP4.5-SSP2 (second panel). These maps were generated using ArcMap 10.3, visit http://desktop.arcgis.com/en/ for more details.

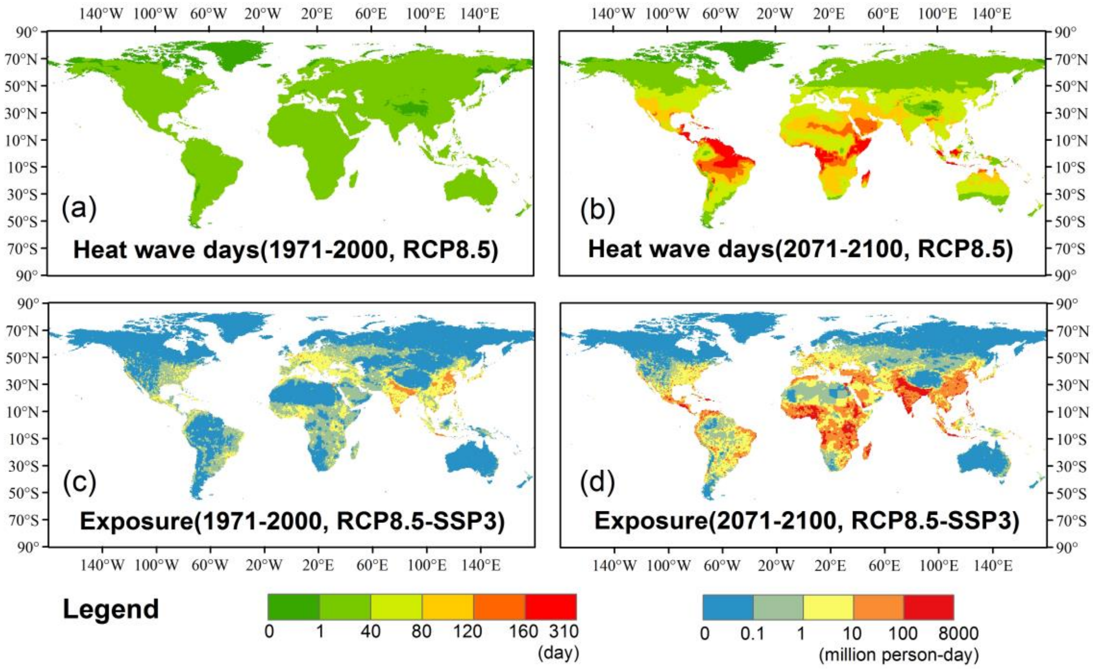

GFDL-ESM2M

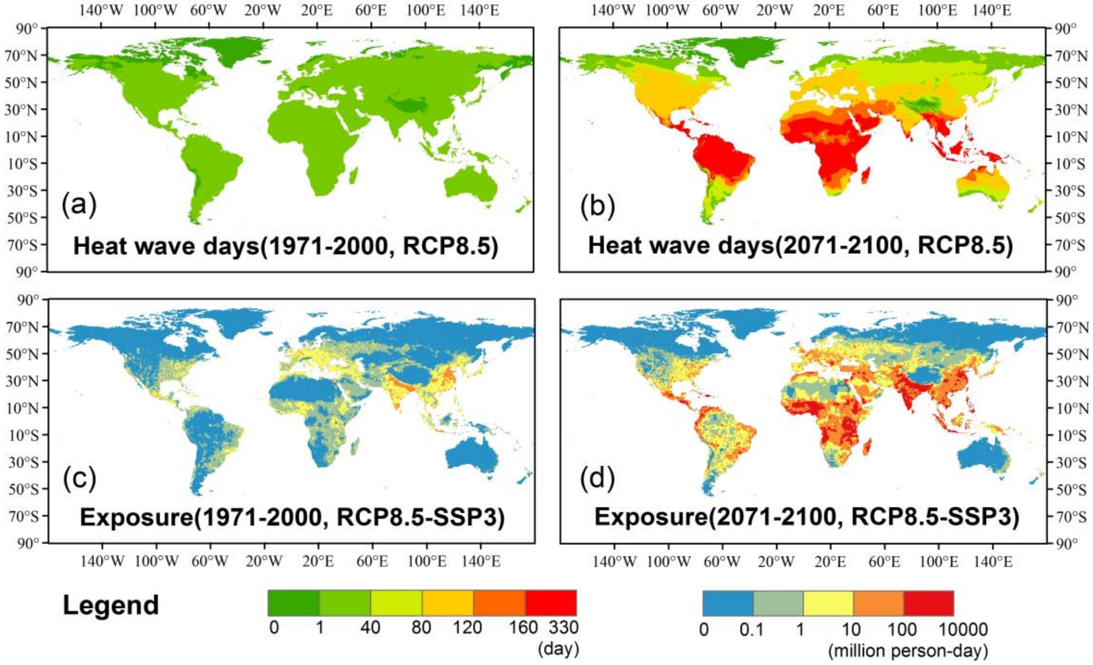

HadGEM2-ES

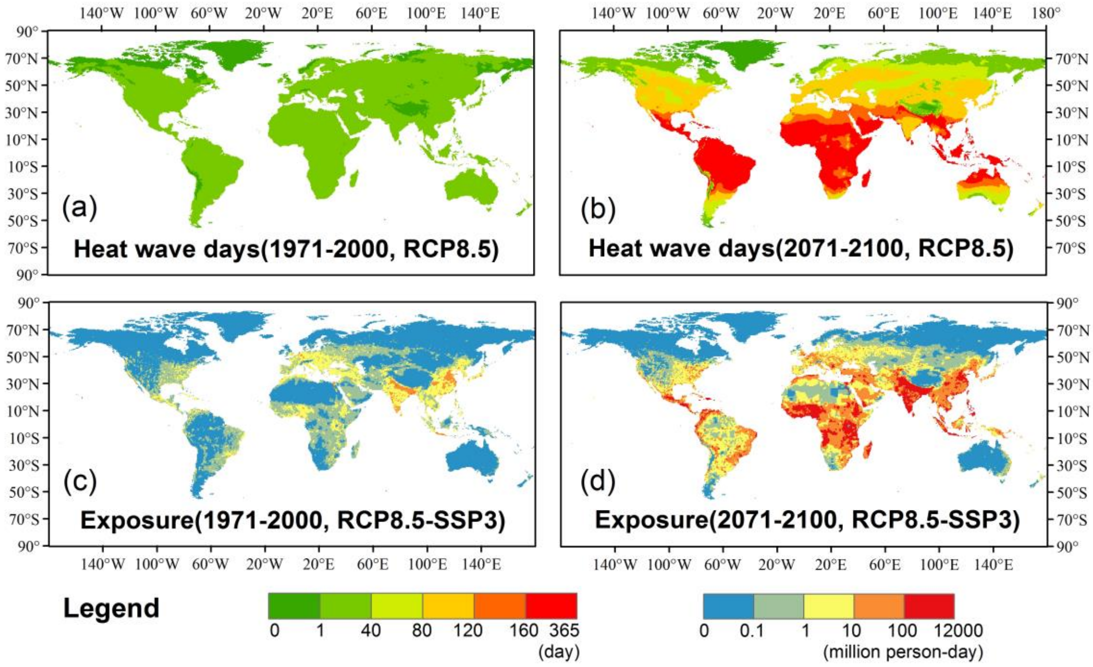

IPSL-CM5A-LR

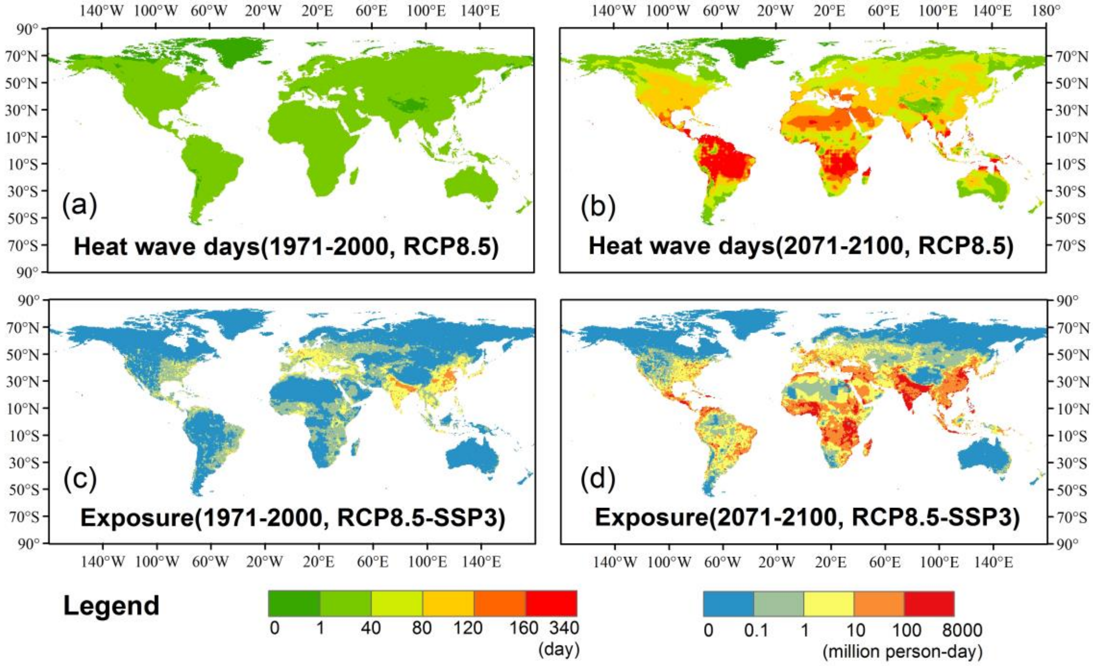

MIROC-ESM-CHEM

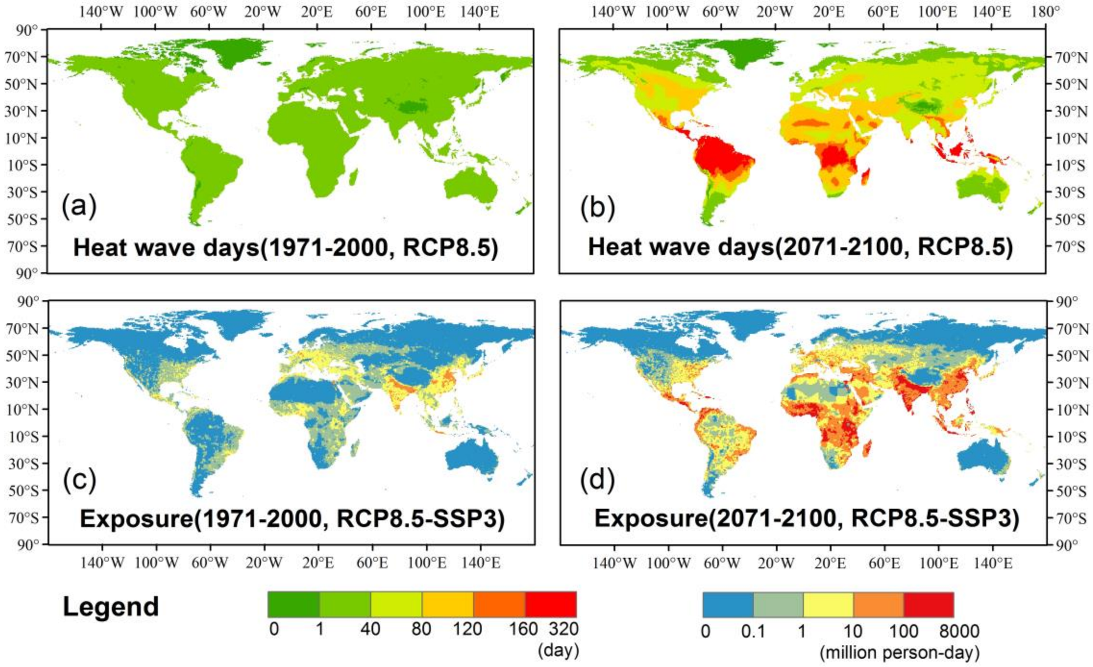

NorESM1-M

Supplementary Figure S4 Heat wave days (a, b; left legend) and exposure (c, d; right legend) averaged for the period 1971-2000 (left: a, c) and the 2071-2100 period (right: b, d) under scenario RCP8.5-SSP3 for five models used in this study. These maps were generated using ArcMap 10.3, visit http://desktop.arcgis.com/en/ for more details.

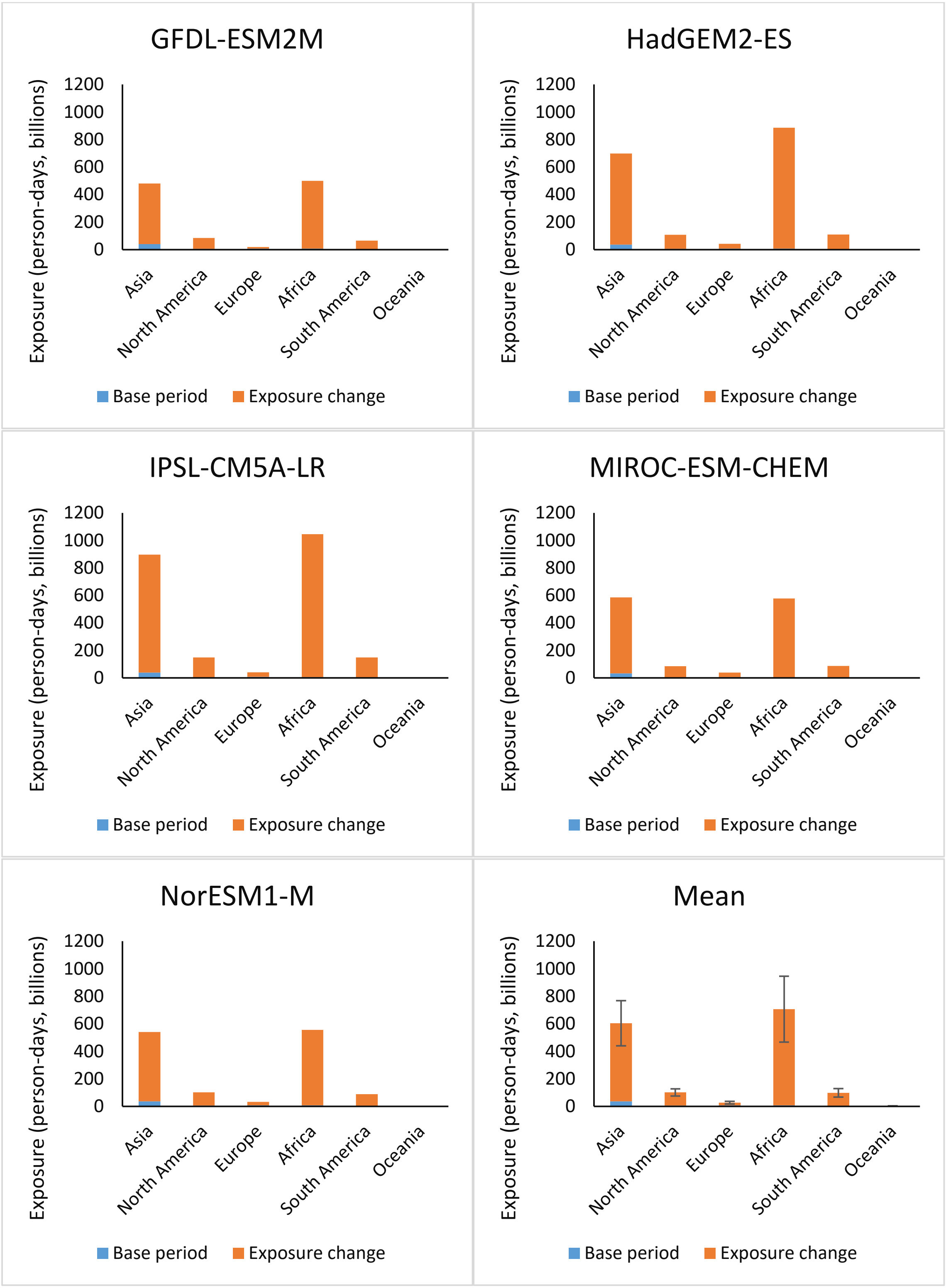

Supplementary Figure S5 Aggregate exposure in the base period (blue) and projected change (orange) under RCP8.5-SSP3 scenario for five models individually and multi-model average, areally aggregated across six continental-scale regions – see Fig.1 in main text for region designations. Error bars illustrate the standard deviation in projected exposure change across the five models.

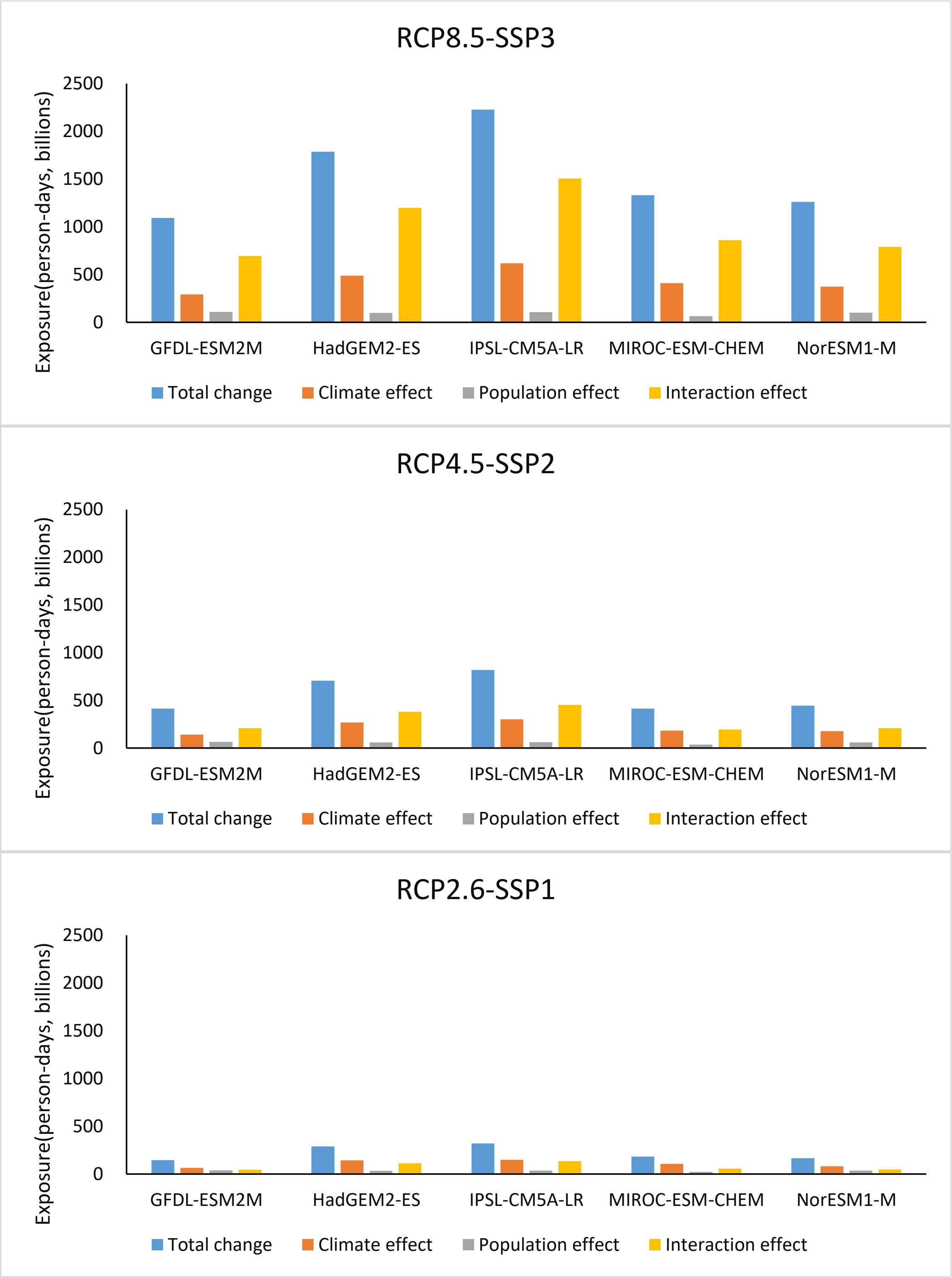

Supplementary Figure S6 Decomposition of aggregate global projected change in exposure under three scenarios for five models used in this study. Increase in total projected exposure change (blue); exposure change from the climate effect (orange) keeping population constant; exposure change from the population effect (gray) keeping climate constant; and exposure change from the interaction effect (yellow) between climate and population – see Methods Section for details.

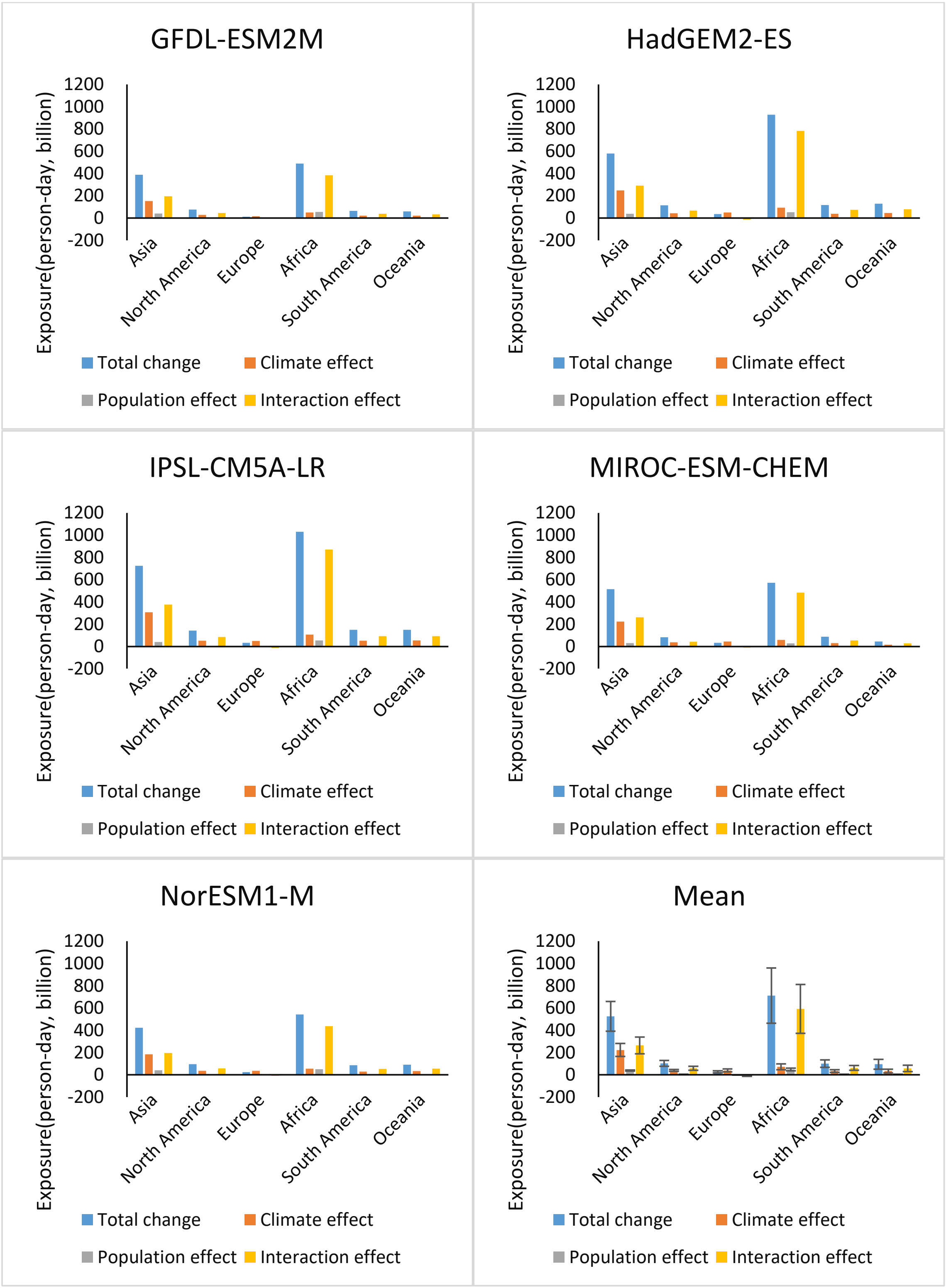

Supplementary Figure S7 Supplementary Figure 6: Decomposition of aggregate regional projected change in exposure under RCP8.5-SSP3 scenario for five models used in this study. Increase in total projected exposure change (blue); exposure change from the climate effect (orange) keeping population constant; exposure change from the population effect (gray) keeping climate constant; and exposure change from the interaction effect (yellow) between climate and population – see Methods Section for details. Error bars illustrate the standard deviation in total projected exposure change across the models for each region/effect.

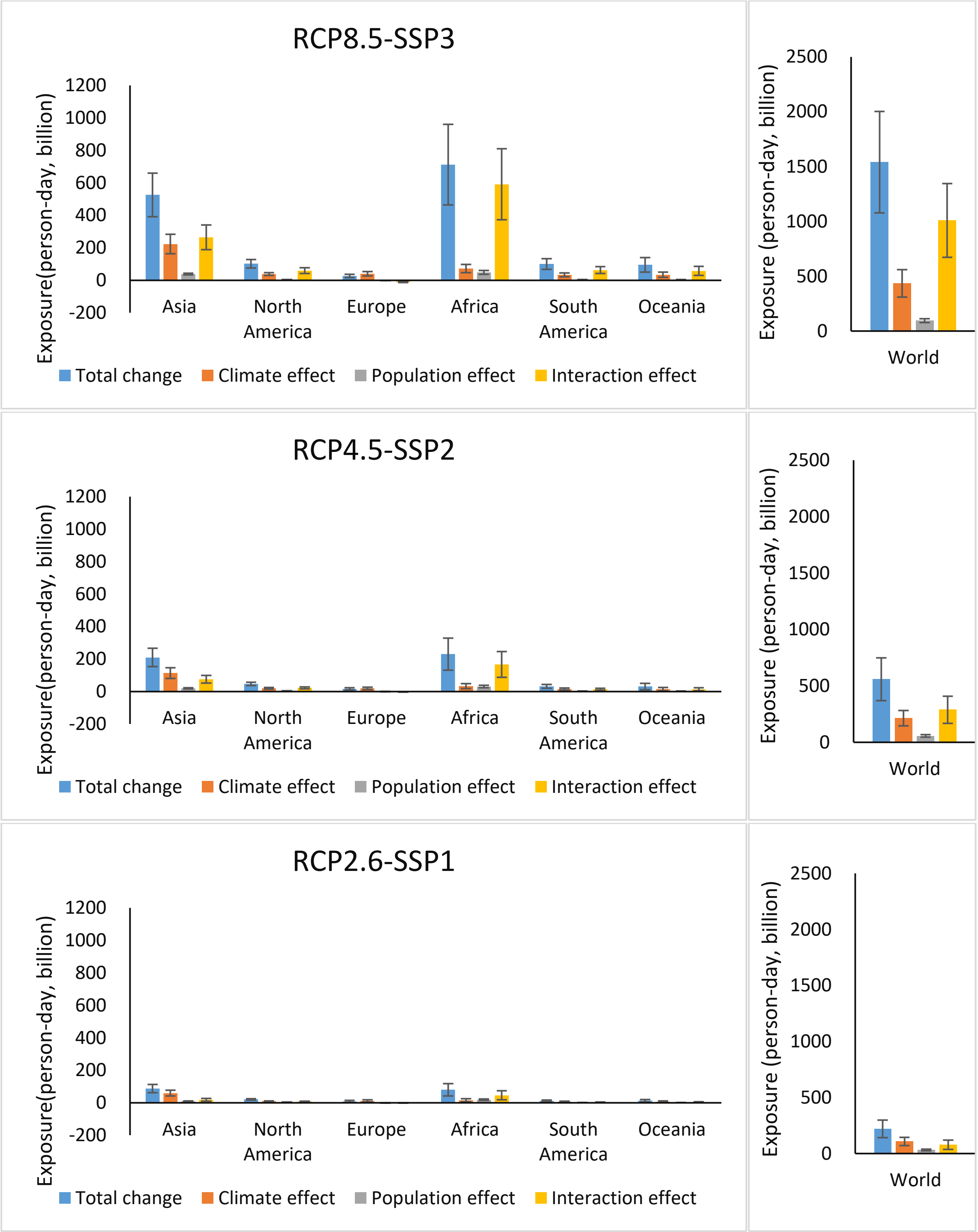

Supplementary Figure S8 Decomposition of aggregate regional (a) and global (b) projected change in exposure under three scenarios. Multi-model average increase in total projected exposure change (blue), exposure change from the climate effect (orange) keeping population constant; exposure change from the population effect (gray) keeping climate constant; and exposure change from the interaction effect (yellow) between climate and population – see Methods Section for details. Error bars illustrate the standard deviation in total projected exposure change across the models for each region/effect.

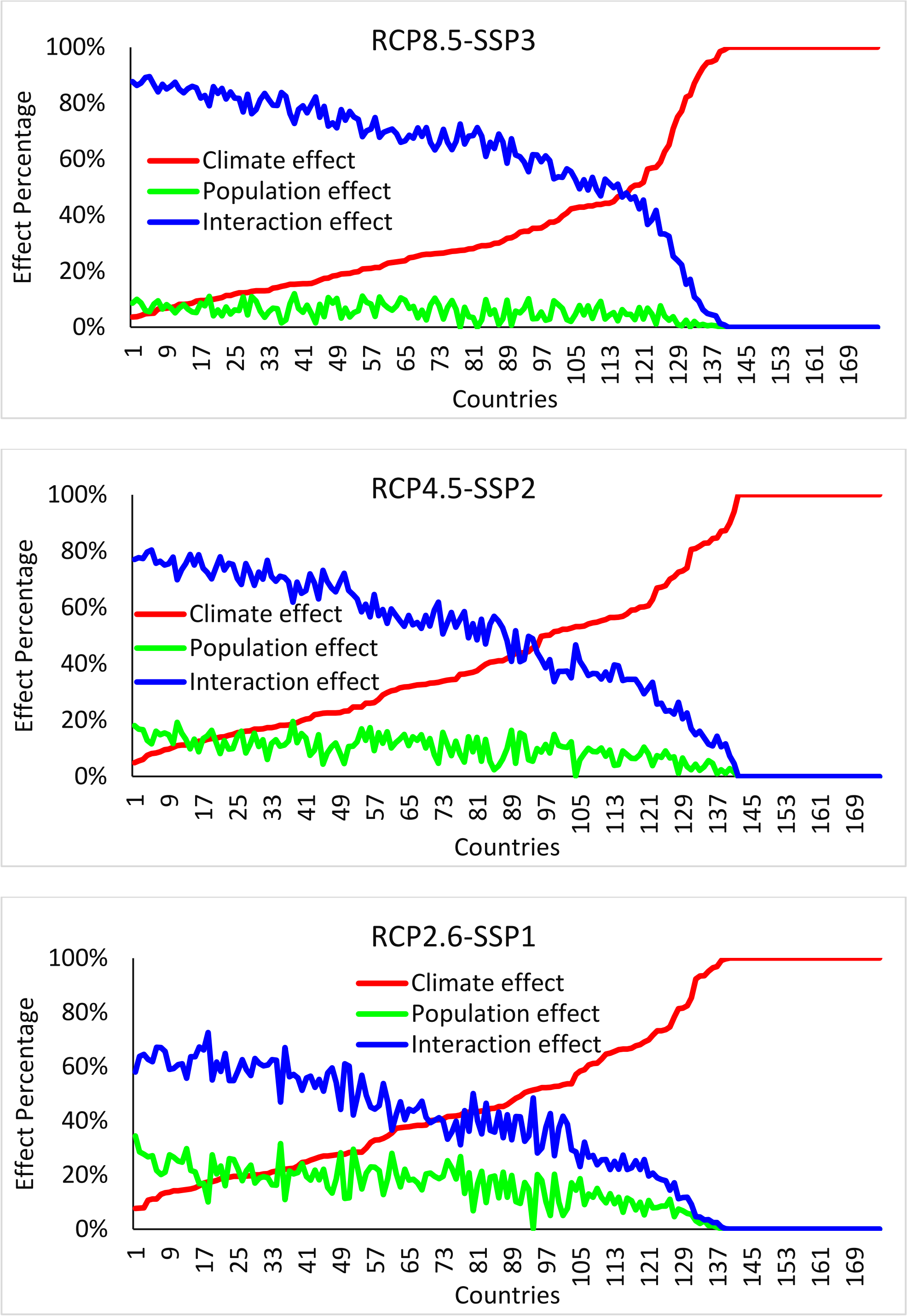

Supplementary Figure S9 Fractional contribution to aggregate country-wide projected change in exposure under RCP8.5-SSP3, RCP4.5-SSP2 and RCP2.6-SSP1 from the climate effect (red), population effect (green) and interaction effect (blue). For clarity countries sorted by the fractional contribution of the climate effect. For countries with negative population growth, climate effect is set to 100%.

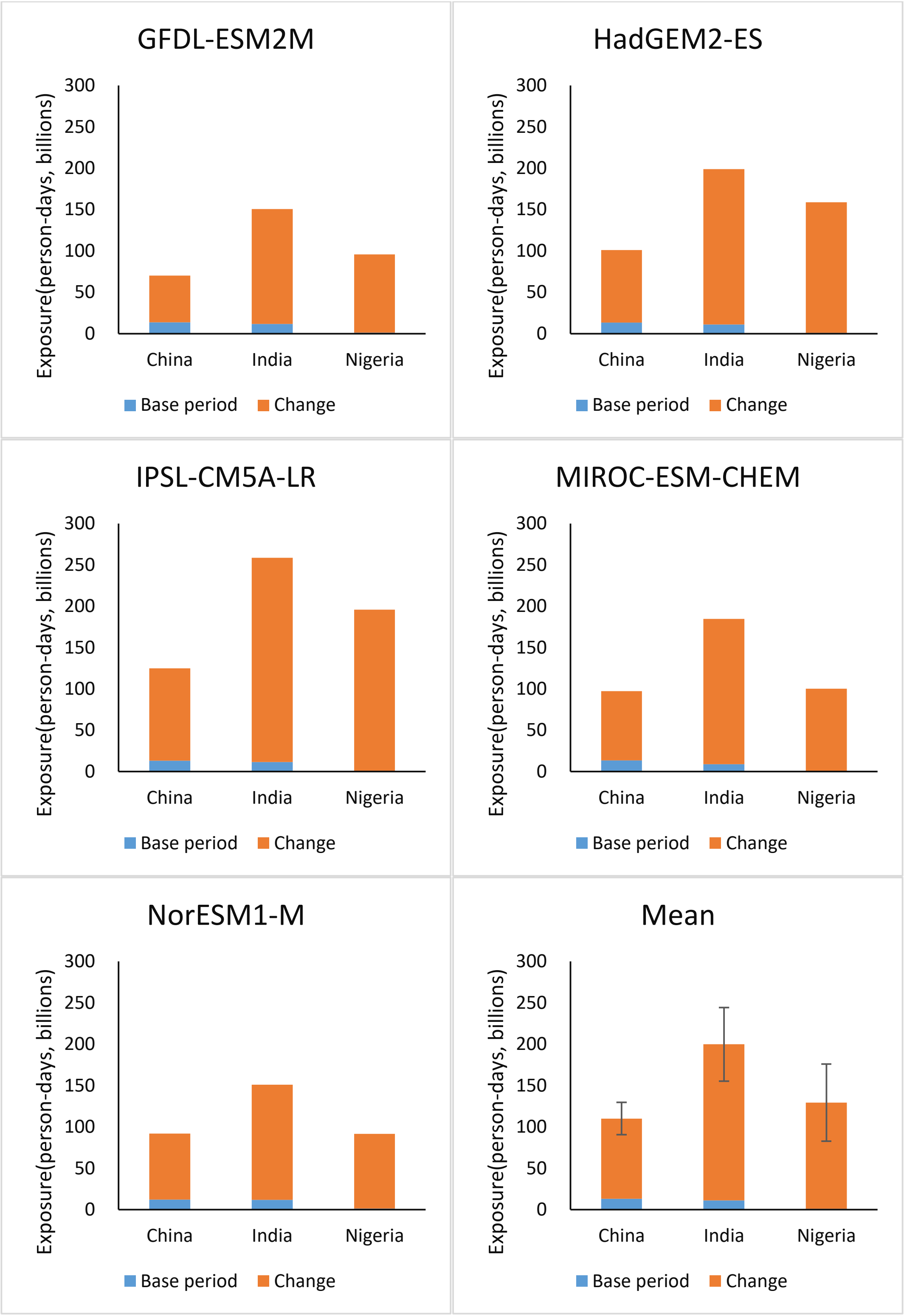

Supplementary Figure S10 Aggregate exposure in the base period (blue) and projected change (orange) under RCP8.5-SSP3 scenario for five models individually and multi-model average for China, India and Nigeria. Error bars illustrate the standard deviation in projected exposure change across the models for each country.

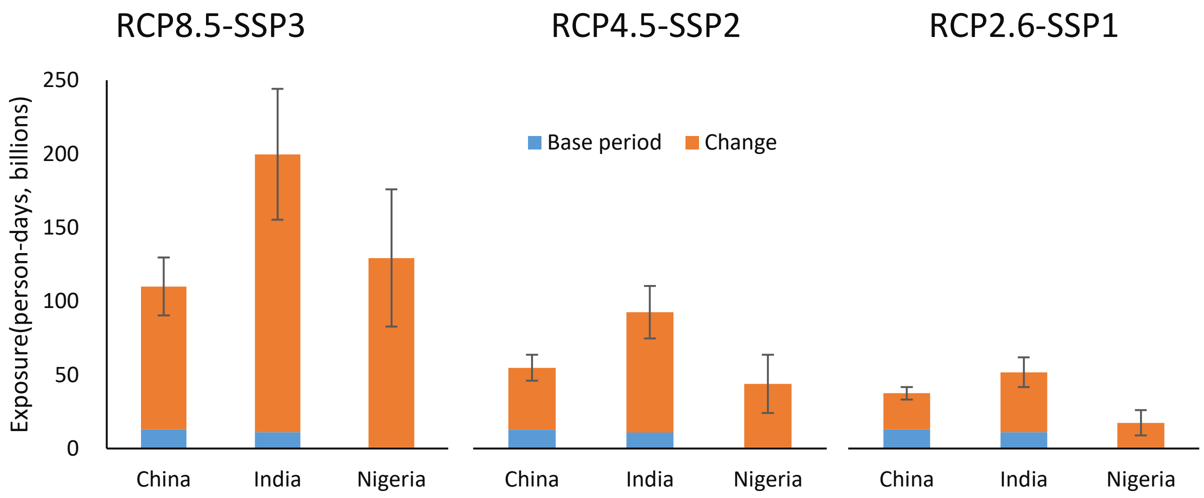

Supplementary Figure S11. Multi-model average of aggregate exposure in the base period (blue) and projected change (orange) for China, India and Nigeria under three emissions scenarios. Error bars illustrate the standard deviation in total projected exposure change across the models for each country.

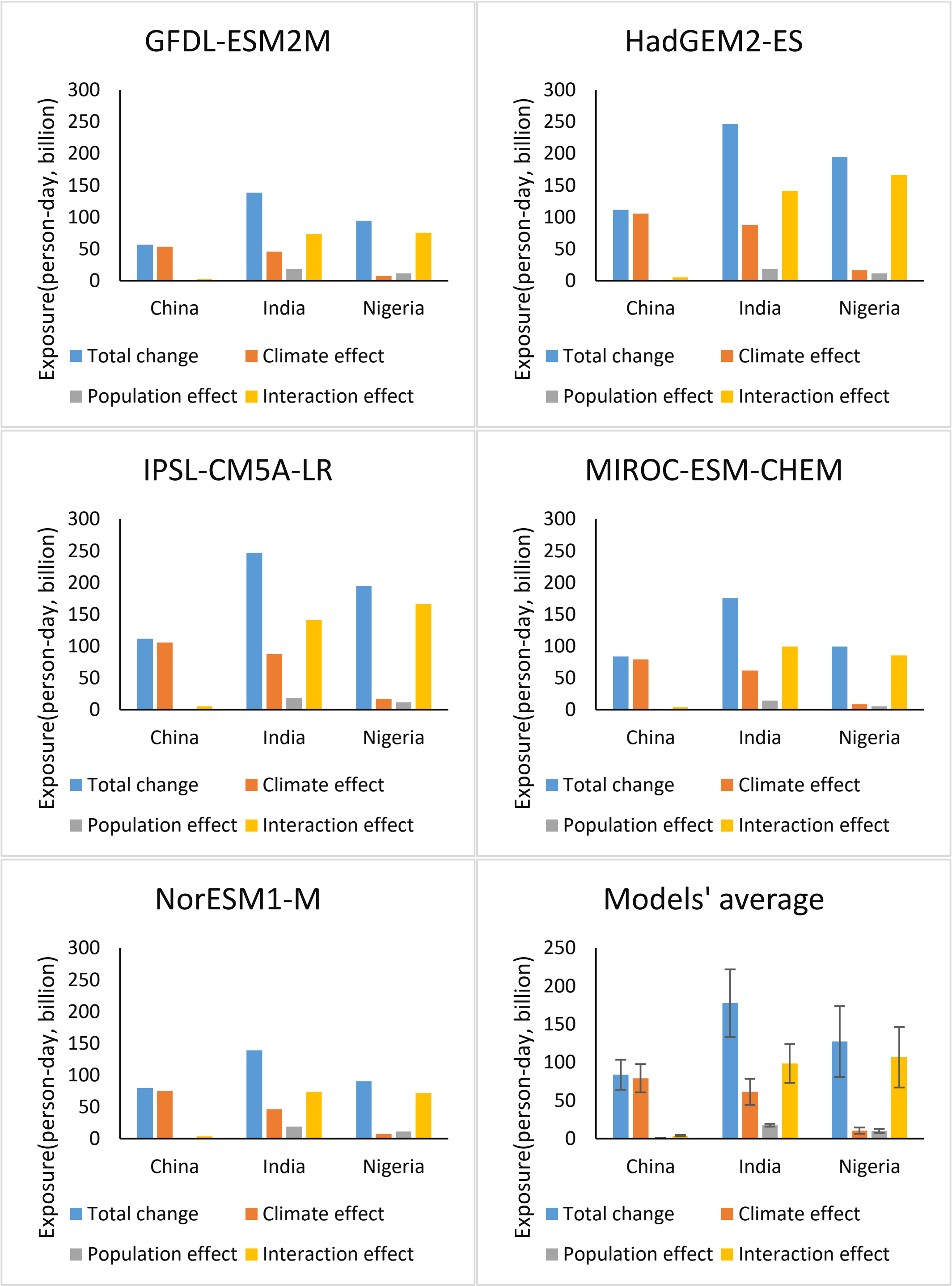

Supplementary Figure S12 Decomposition of aggregate country-level projected change in exposure for China, India, and Nigeria under three emissions scenarios. Increase in total projected exposure change (blue), exposure change from the climate effect (orange) keeping population constant; exposure change from the population effect (gray) keeping climate constant; and exposure change from the interaction effect (yellow) between climate and population – see Methods Section for details. Error bars illustrate the standard deviation in projected exposure change across the models for each country.

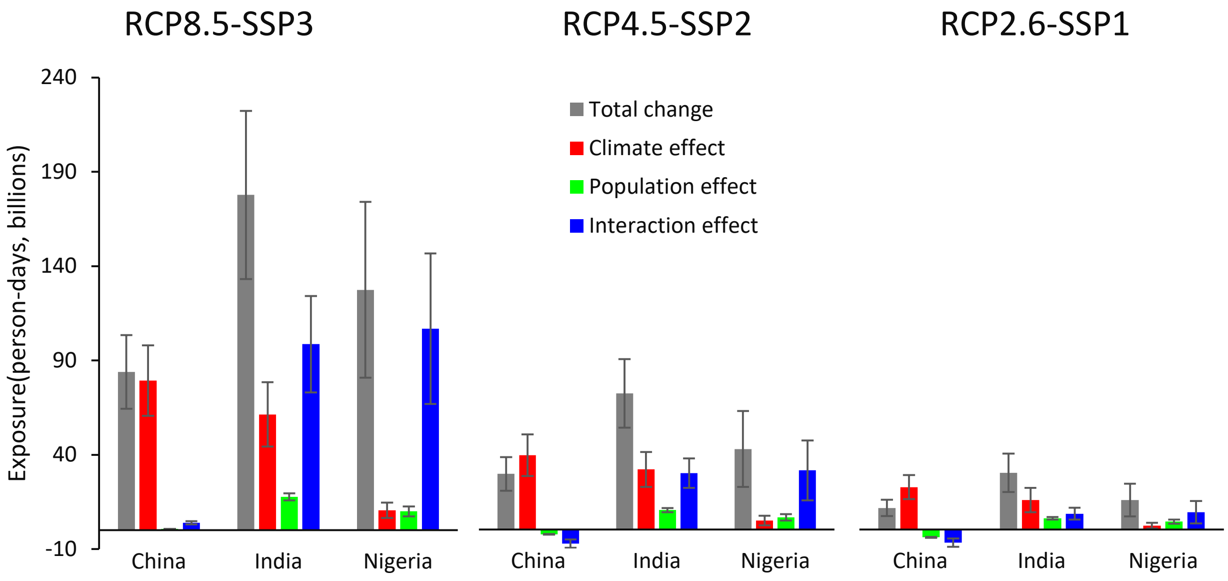

Supplementary Figure S13. Decomposition of aggregate country-level projected change in exposure for China, India, and Nigeria under three emissions scenarios. Multi-model average increase in total projected exposure change (gray), exposure change from the climate effect (red) keeping population constant; exposure change from the population effect (green) keeping climate constant; and exposure change from the interaction effect (blue) between climate and population – see Methods Section for details. Error bars illustrate the standard deviation in total projected exposure change across the models for each country/effect.

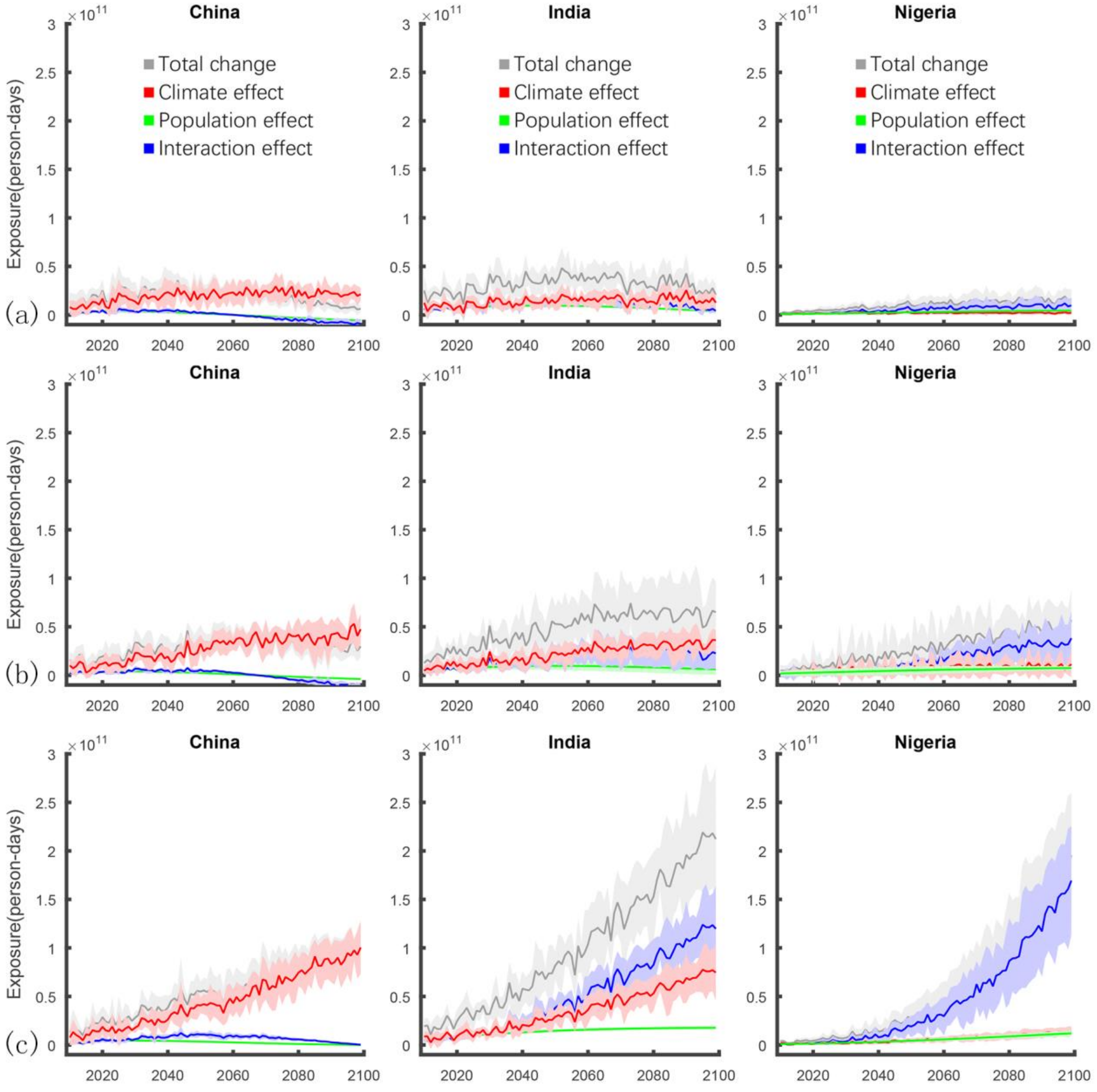

Supplementary Figure S14. Decomposition of aggregate country-level projected change in exposure for China, India, and Nigeria under three emissions scenarios yearly (Row (a): RCP2.6-SSP1; Row (b): RCP4.5-SSP2; Row (c):RCP-SSP3). Multi-model average increase in total projected exposure change (gray), exposure change from the climate effect (red) keeping population constant; exposure change from the population effect (green) keeping climate constant; and exposure change from the interaction effect (blue) between climate and population – see Methods Section for details. Shadows illustrate the standard deviation in total projected exposure change across the models for each country/effect.

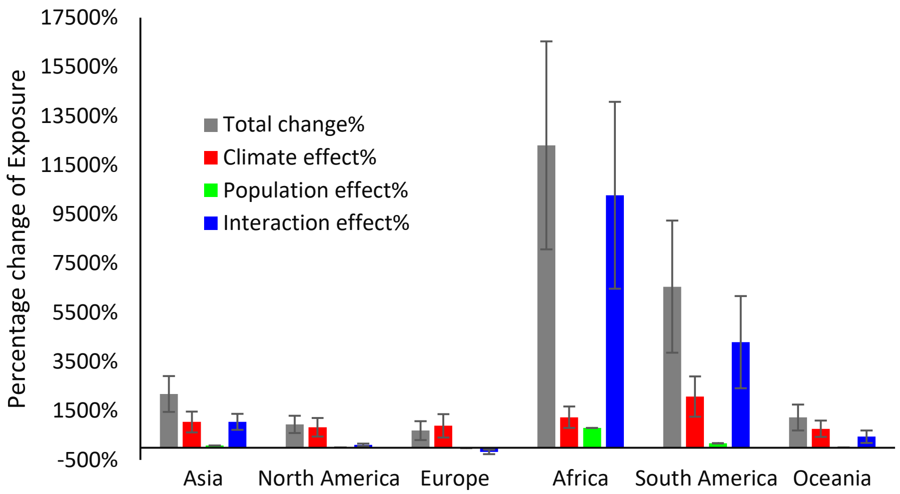

Supplementary Figure S15 Percentage increase in aggregate regional exposure under RCP8.5-SSP3. Multi-model average increase in total exposure change (gray), exposure change from the climate effect (red) keeping population constant; exposure change from the population effect (green) keeping climate constant; and exposure change from the interaction effect (blue) between climate and population – see Methods Section for details. Error bars illustrate the standard deviation in total projected exposure change across the models for each region/effect.

95th percentile threshold, HWD as hazard

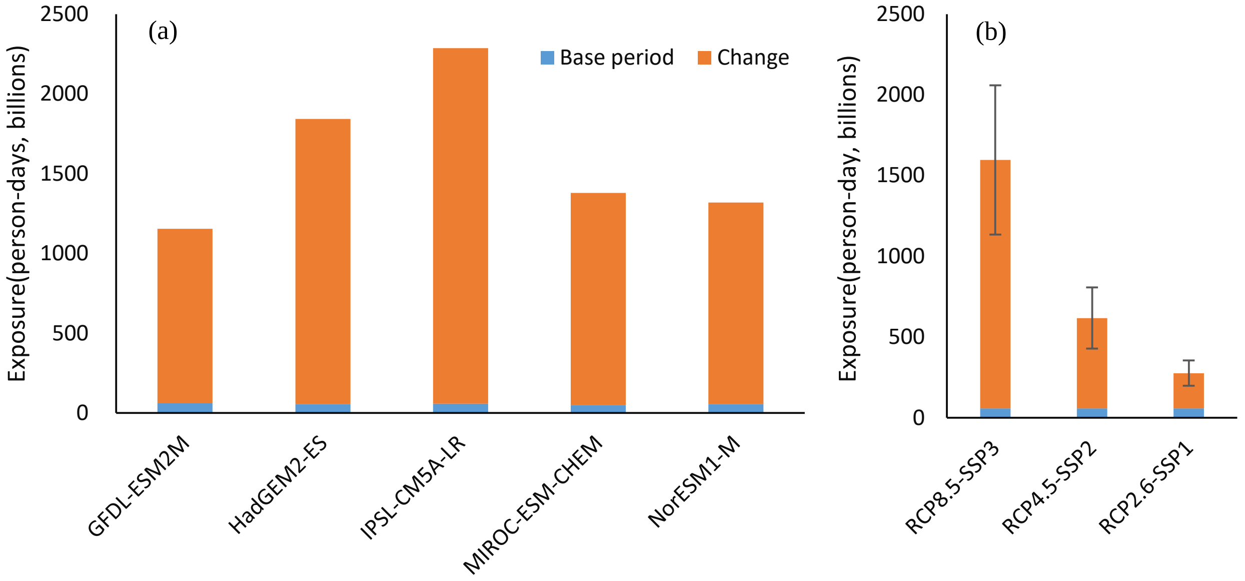

97.5th percentile threshold, HWD as hazard

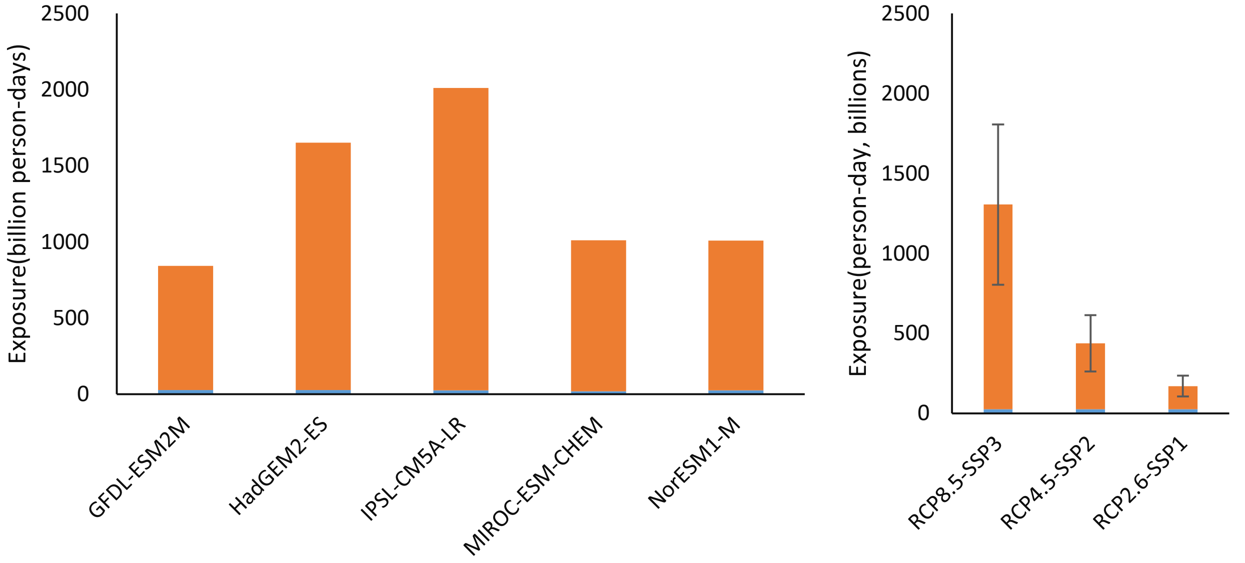

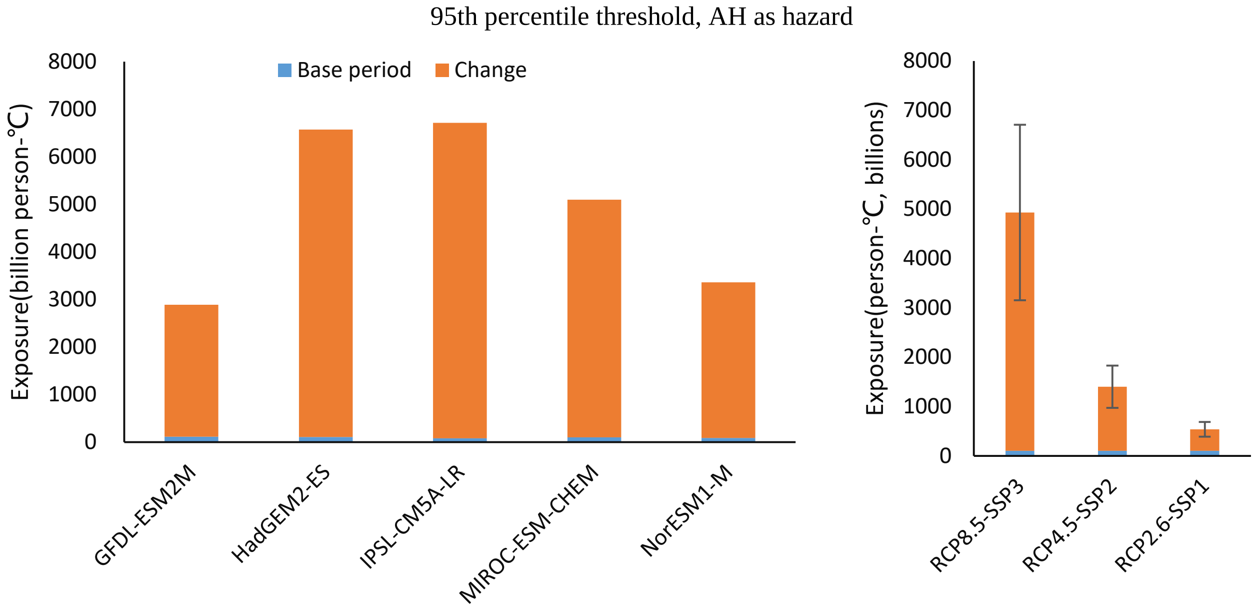

Supplementary Figure S16. Column (b) Multi-model average of global aggregate exposure under three scenario combinations. Error bars illustrate the standard deviation in projected exposure change across the models. Column (a) Global aggregate exposure in the base period (blue) and projected change (orange) under RCP8.5-SSP3 scenario for five models used in this study. First row is the results with 95th percentile HWD as hazard; second row is used 97.5th percentile HWD as hazard; third row is the results with 95th percentile AH as hazard.

95th percentile threshold, HWD as hazard

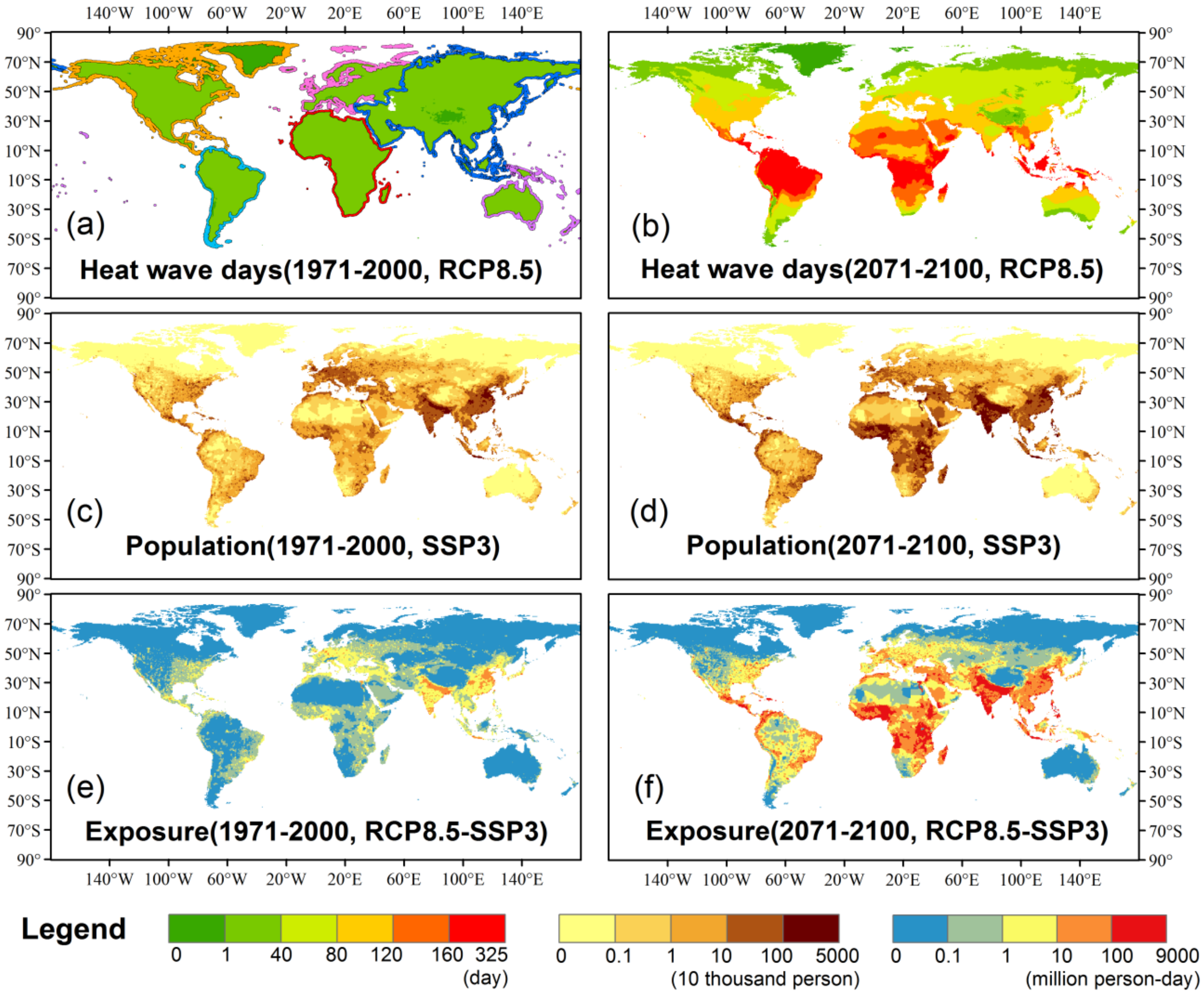

97.5th percentile threshold, HWD as hazard

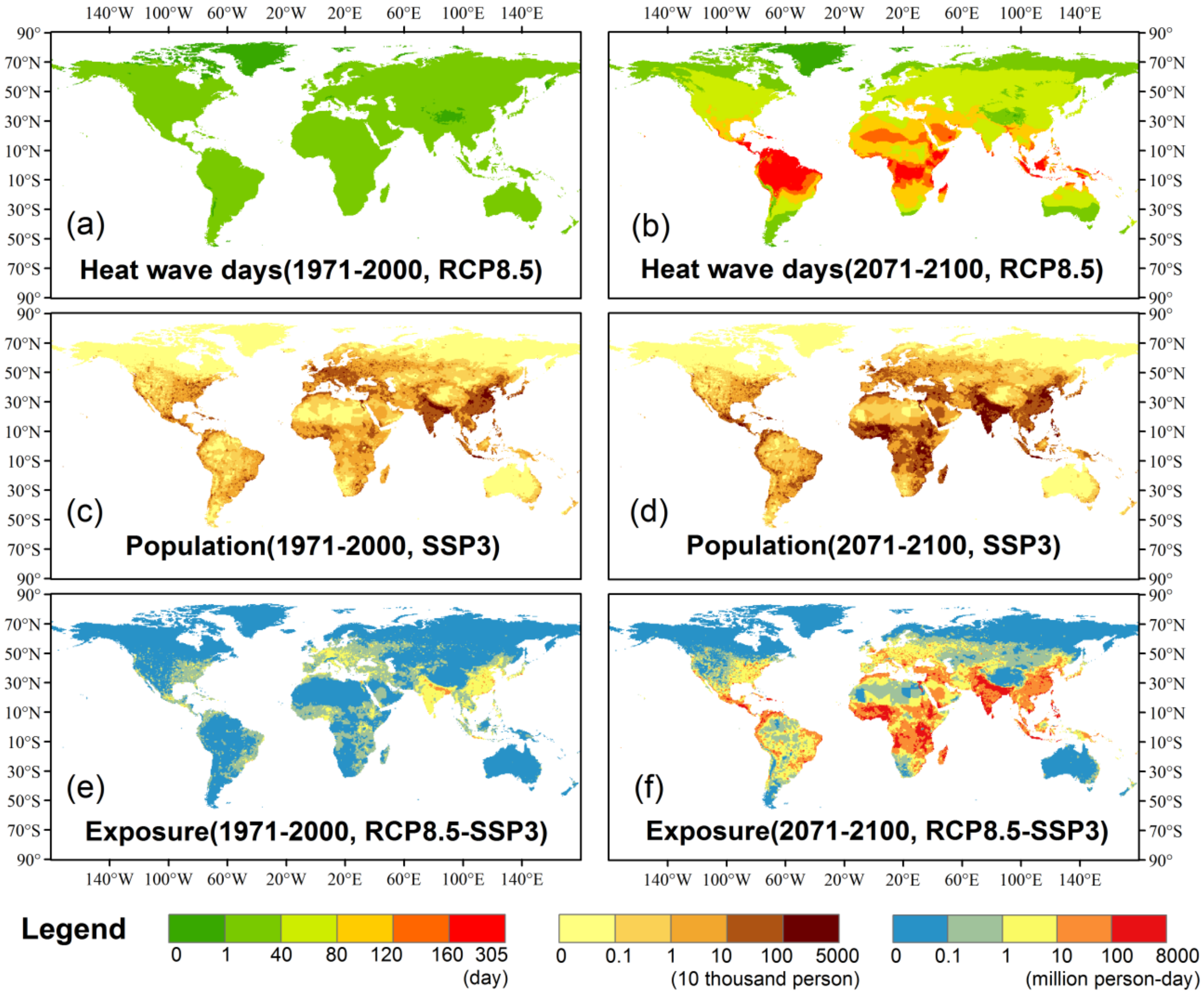

95th percentile threshold, AH as hazard

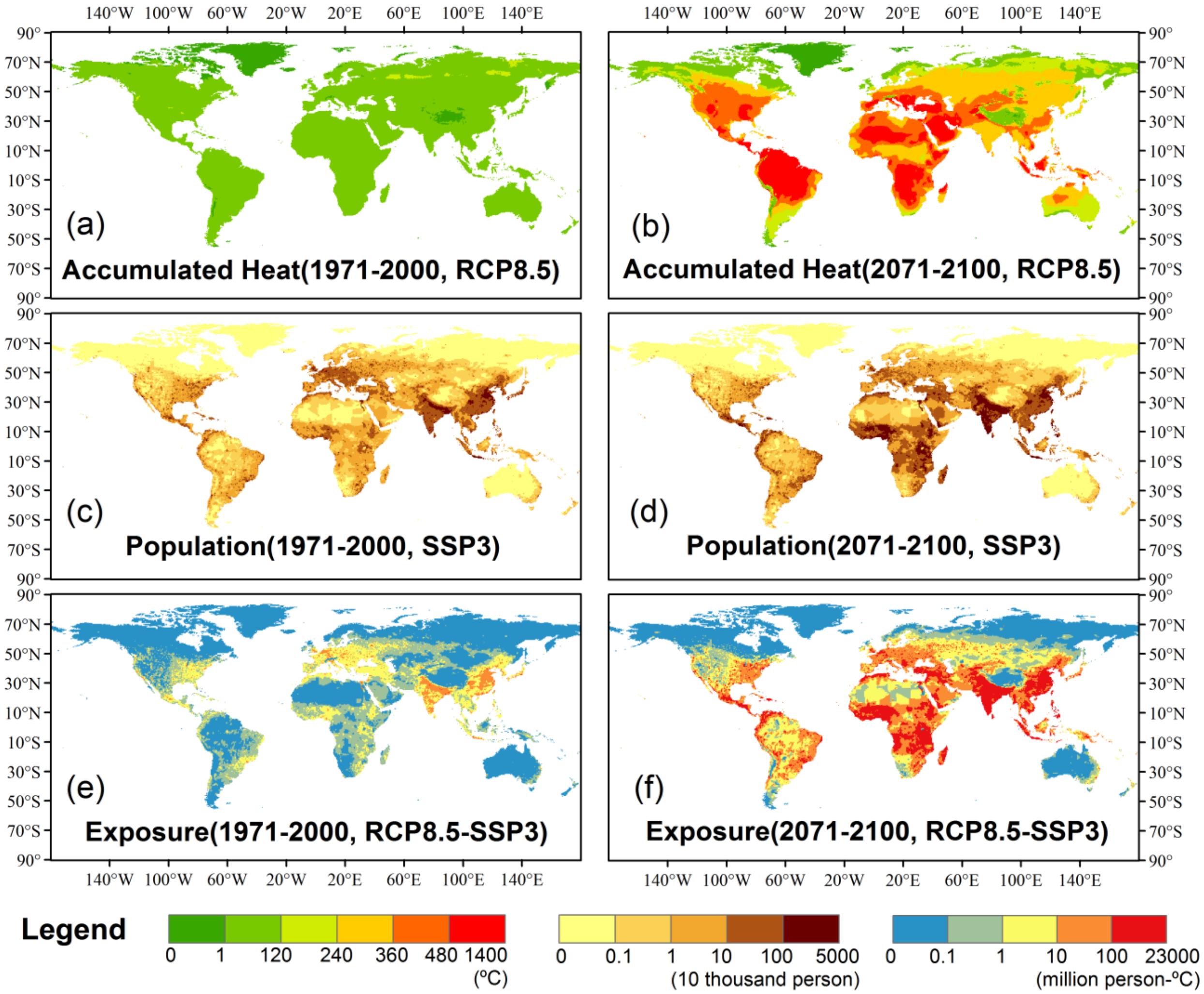

Supplementary Figure S17. Multi-model average of heat wave days/accumulated heat (a, b; left legend), population (c, d; middle legend), and exposure (e, f; right legend) averaged for the period 1971-2000 (left: a, c, e) and the 2071-2100 period (right: b, d, f) under scenario RCP8.5-SSP3. Boundaries of six colors in (e,f) outline the continental regions analyzed below. First panel is the results with 95th percentile HWD as hazard; second panel is used 97.5th percentile HWD as hazard; third panel is the results with 95th percentile AH as hazard. These maps were generated using ArcMap 10.3, visit http://desktop.arcgis.com/en/ for more details.

95th percentile threshold, HWD as hazard

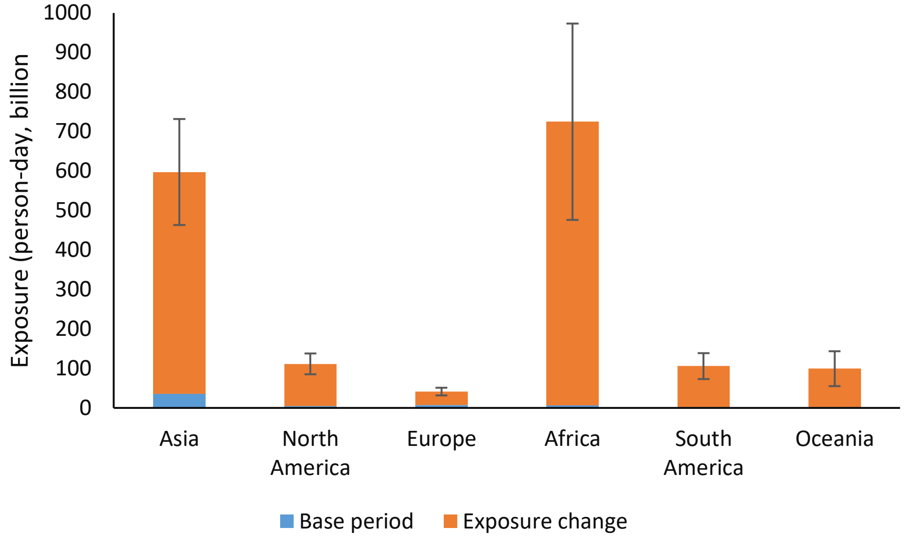

97.5th percentile threshold, HWD as hazard

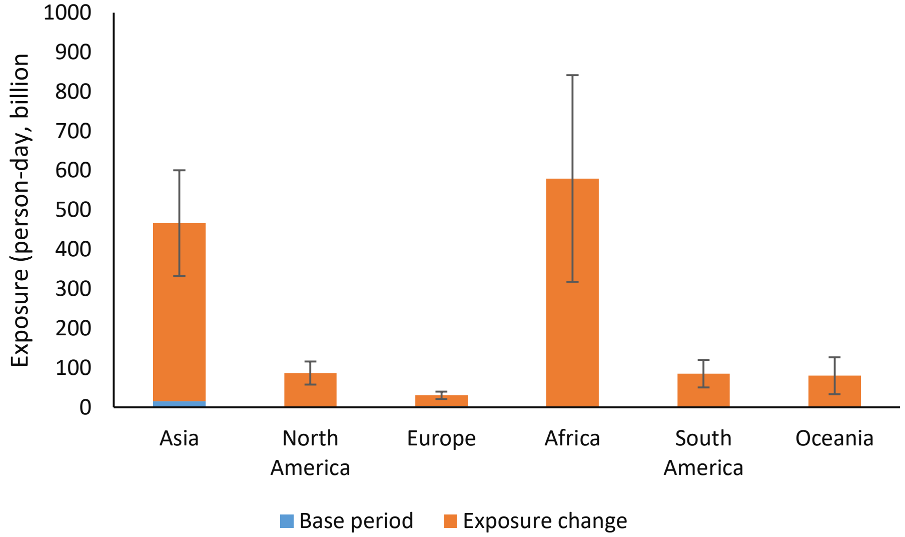

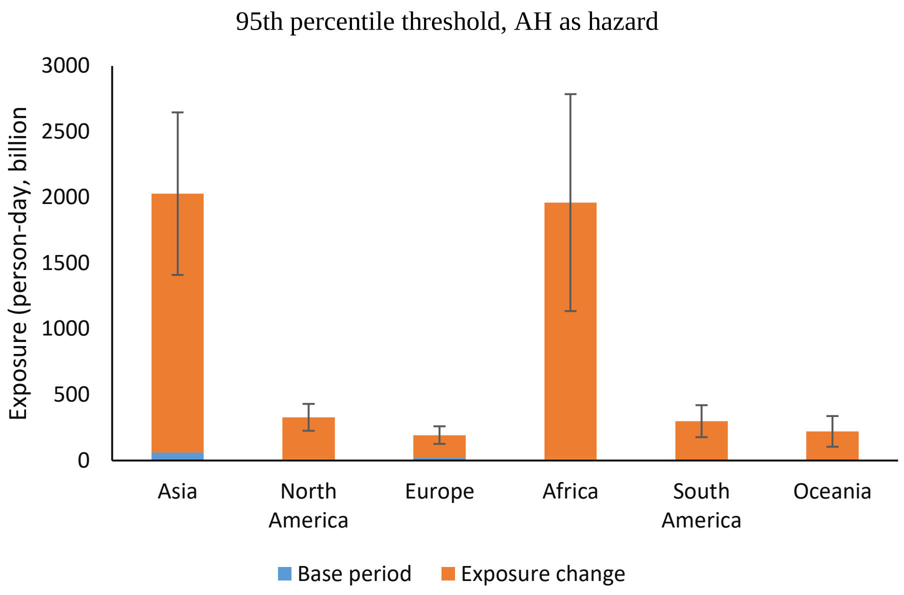

Supplementary Figure S18. Multi-model average of aggregate exposure in the base period (blue) and projected change (orange) under RCP8.5-SSP3, areally aggregated across six continental-scale regions – see Fig.2 for region designations. Error bars illustrate the standard deviation in projected exposure change across the five models. First row is the results with 95th percentile HWD as hazard; second row is used 97.5th percentile HWD as hazard; third row is the results with 95th percentile AH as hazard.

95th percentile threshold, HWD as hazard

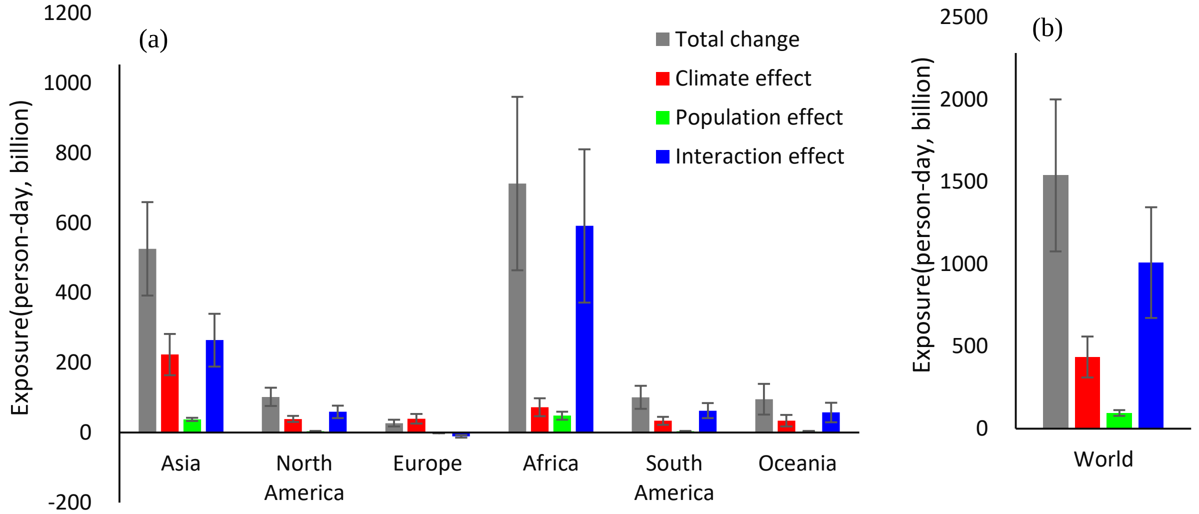

97.5th percentile threshold, HWD as hazard

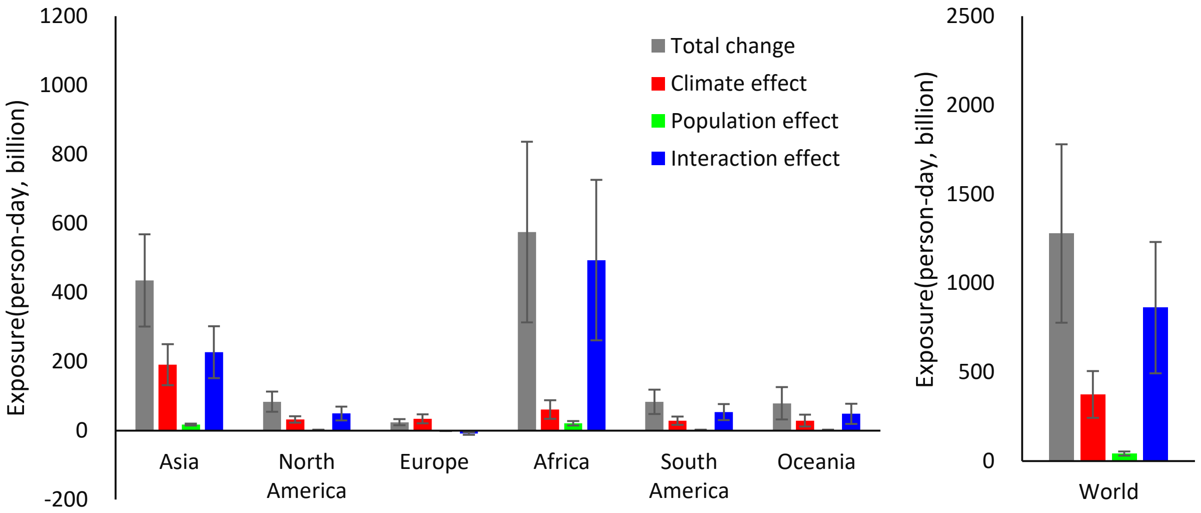

95th percentile threshold, AH as hazard

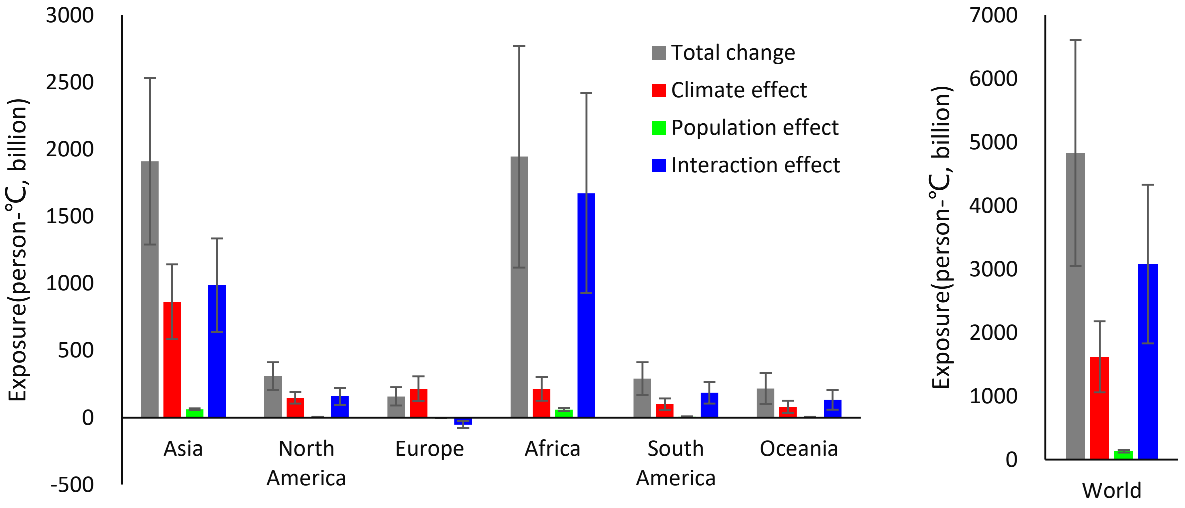

Supplementary Figure S19. Decomposition of aggregate regional (column a) and global (column b) projected change in exposure under RCP8.5-SSP3 scenario. Multi-model average increase in total projected exposure change (gray), exposure change from the climate effect (red) keeping population constant; exposure change from the population effect (green) keeping climate constant; and exposure change from the interaction effect (blue) between climate and population – see Methods Section for details. Error bars illustrate the standard deviation in total projected exposure change across the models for each region/effect. First row is the results with 95th percentile HWD as hazard; second row is used 97.5th percentile HWD as hazard; third row is the results with 95th percentile AH as hazard.

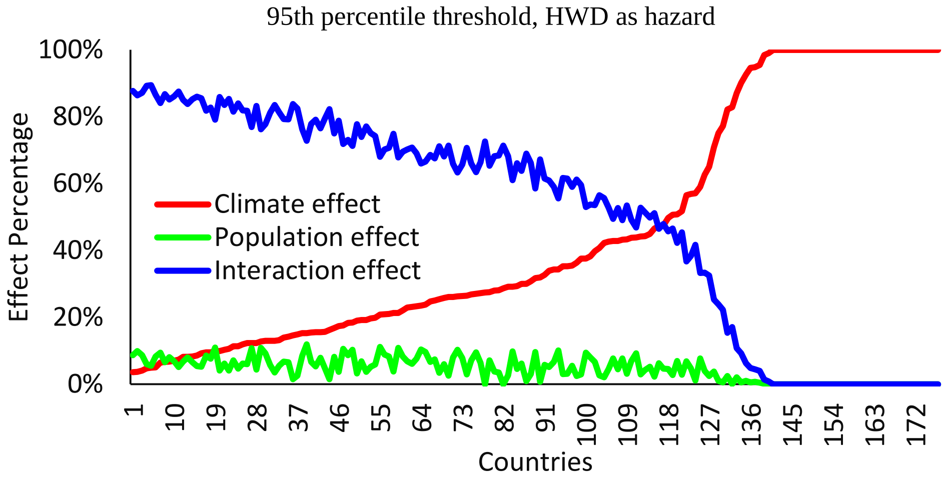

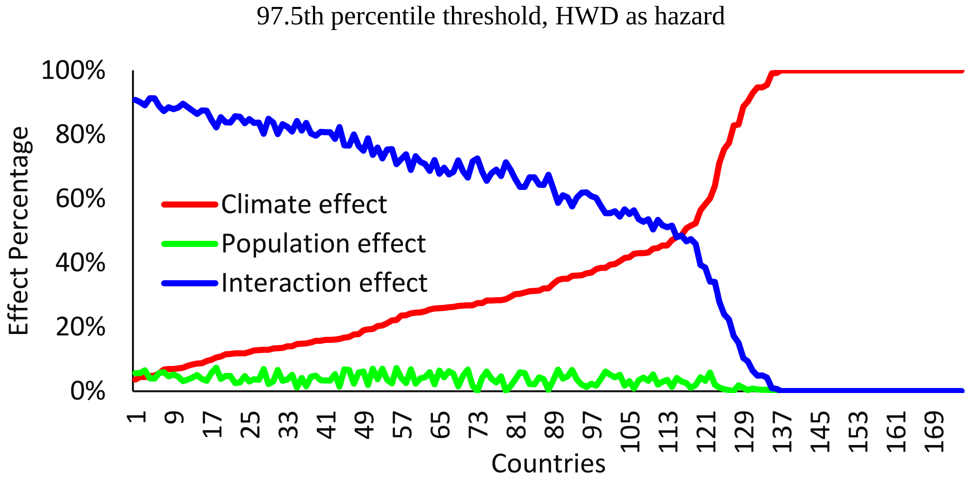

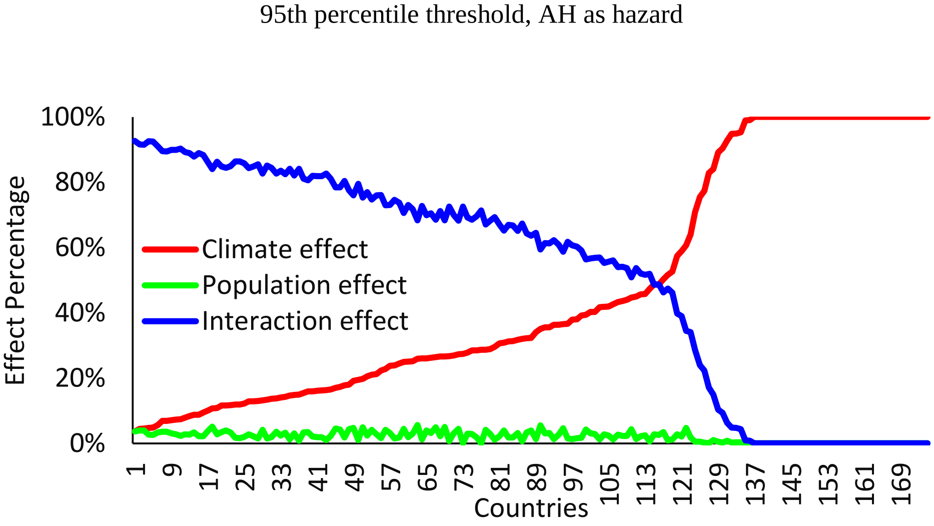

Supplementary Figure S20. Fractional contribution to aggregate country-wide projected change in exposure under RCP8.5-SSP3 scenario from the climate effect (red), population effect (green) and interaction effect (blue). For clarity countries sorted by the fractional contribution of the climate effect. For countries with negative population growth, climate effect is set to 100%. First

row is the results with 95th percentile HWD as hazard; second row is used 97.5th percentile HWD as hazard; third row is the results with 95th percentile AH as hazard.

95th percentile threshold, HWD as hazard

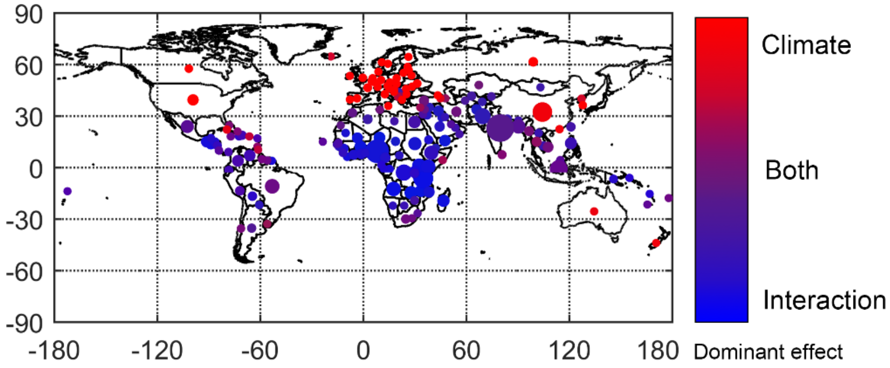

97.5th percentile threshold, HWD as hazard

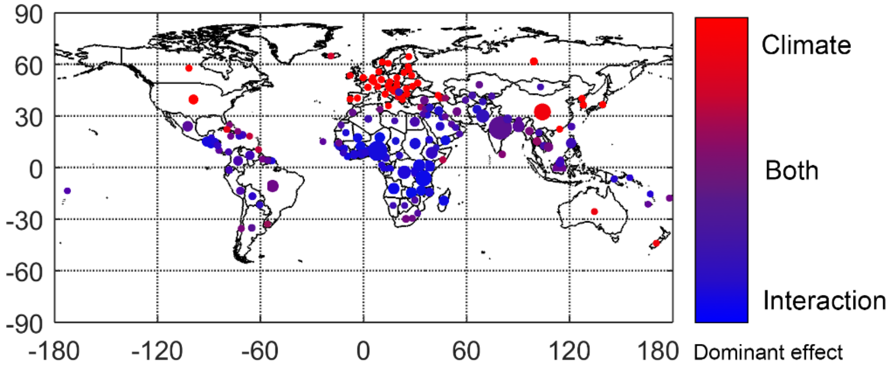

95th percentile threshold, AH as hazard

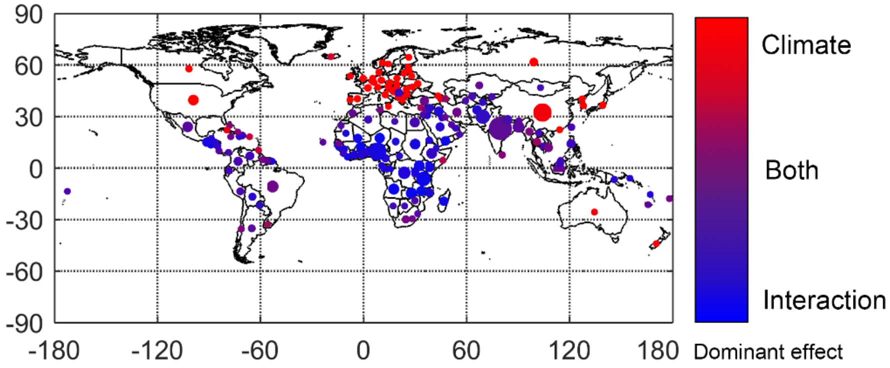

Supplementary Figure S21. Decomposition of aggregate country level projected change in exposure for RCP8.5-SSP3 scenario. The size of the circle refers to total change. Red shading is proportional to the fractional contribution from the climate effect and blue shading is proportional to the fractional contribution from the interaction effect. The fractional contribution from the population effect is also shown and is represented by green shading but for clarity green is not included on the color bar because the effect is small everywhere. First row is the results with 95th percentile HWD as hazard; second row is used 97.5th percentile HWD as hazard; third row is the results with 95th percentile AH as hazard. These maps were generated using Matlab 2015a, visit http://www.mathworks.com/ for more details.

95th percentile threshold, HWD as hazard

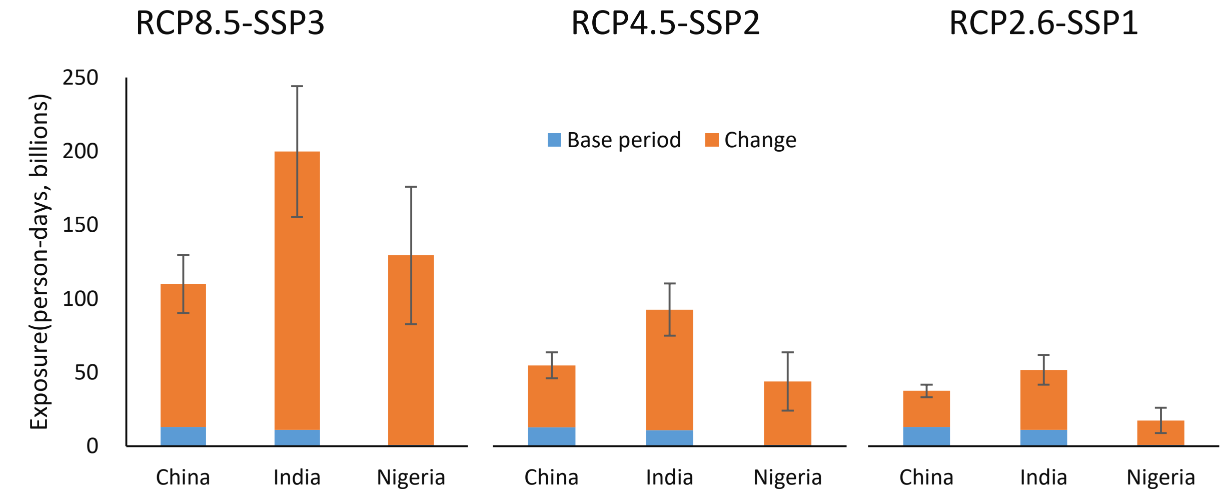

97.5th percentile threshold, HWD as hazard

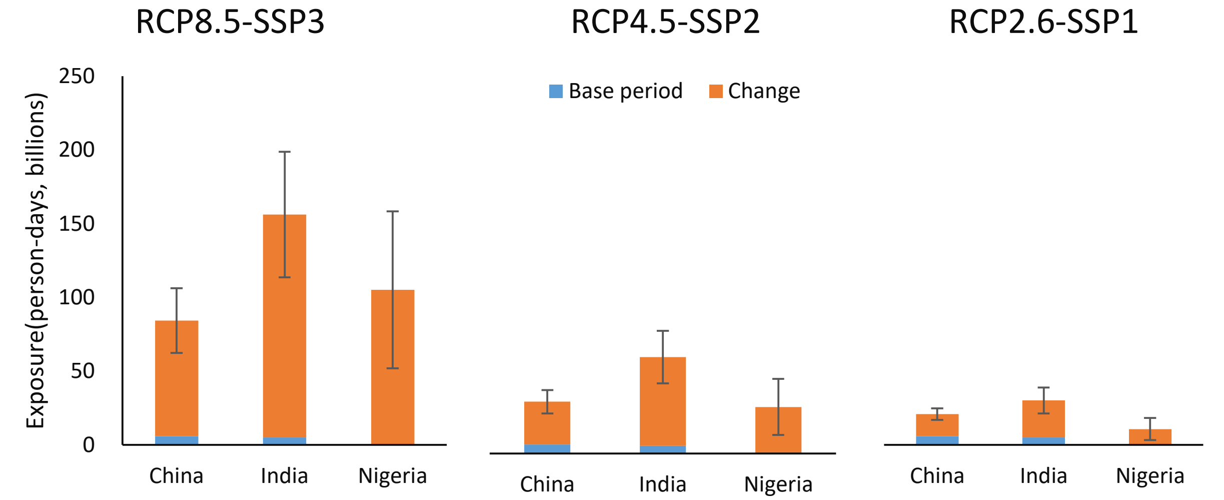

95th percentile threshold, AH as hazard

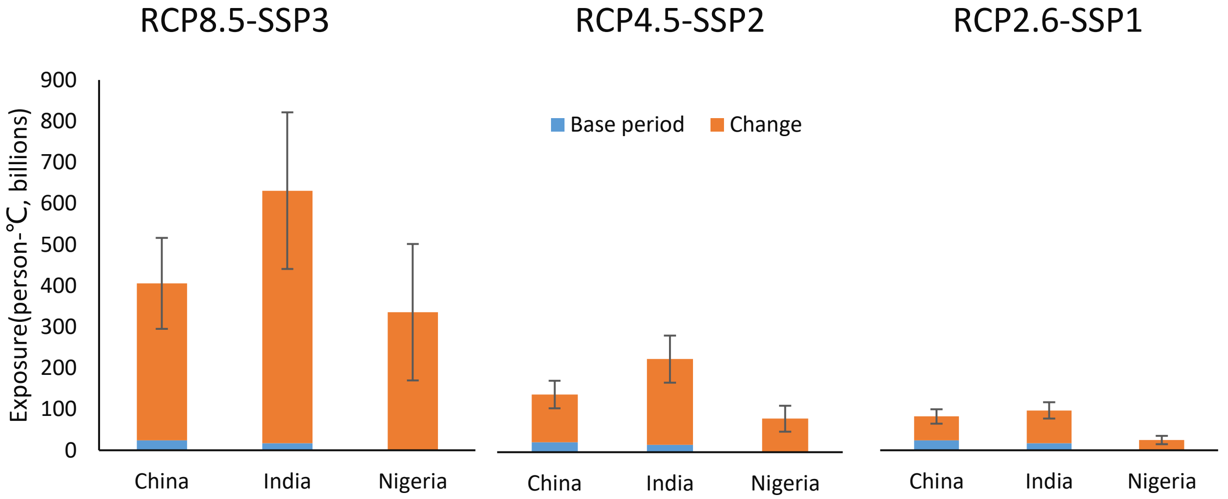

Supplementary Figure S22. Multi-model average of aggregate exposure in the base period (blue) and projected change (orange) for China, India and Nigeria under three emissions scenarios. Error bars illustrate the standard deviation in total projected exposure change across the models for each country. First row is the results with 95th percentile HWD as hazard; second row is used 97.5th percentile HWD as hazard; third row is the results with 95th percentile AH as hazard.

95th percentile threshold, HWD as hazard

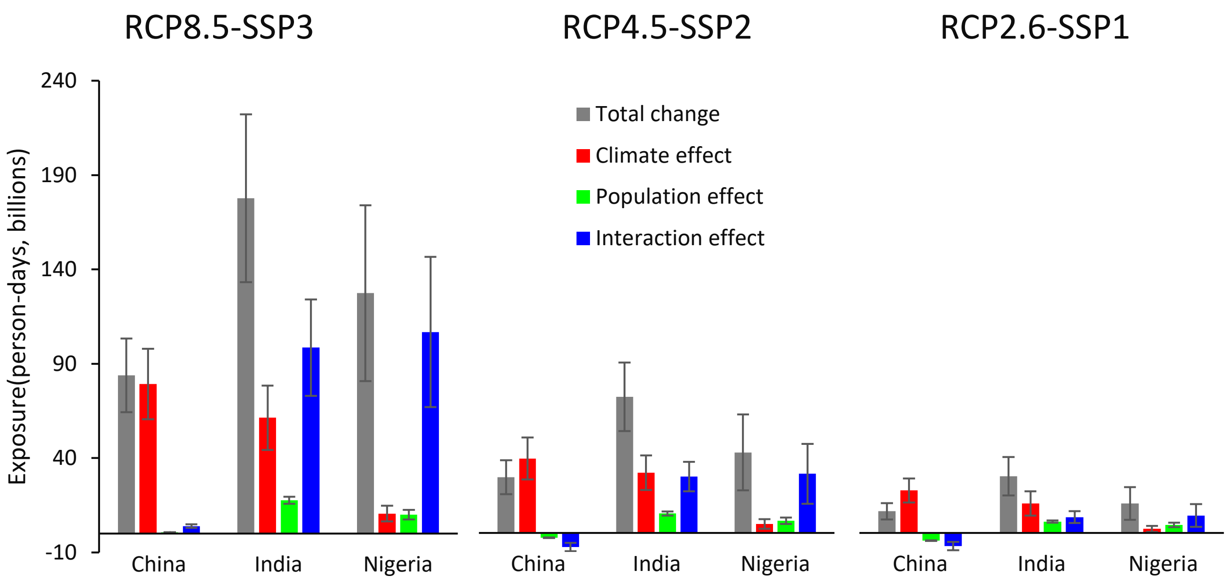

97.5th percentile threshold, HWD as hazard

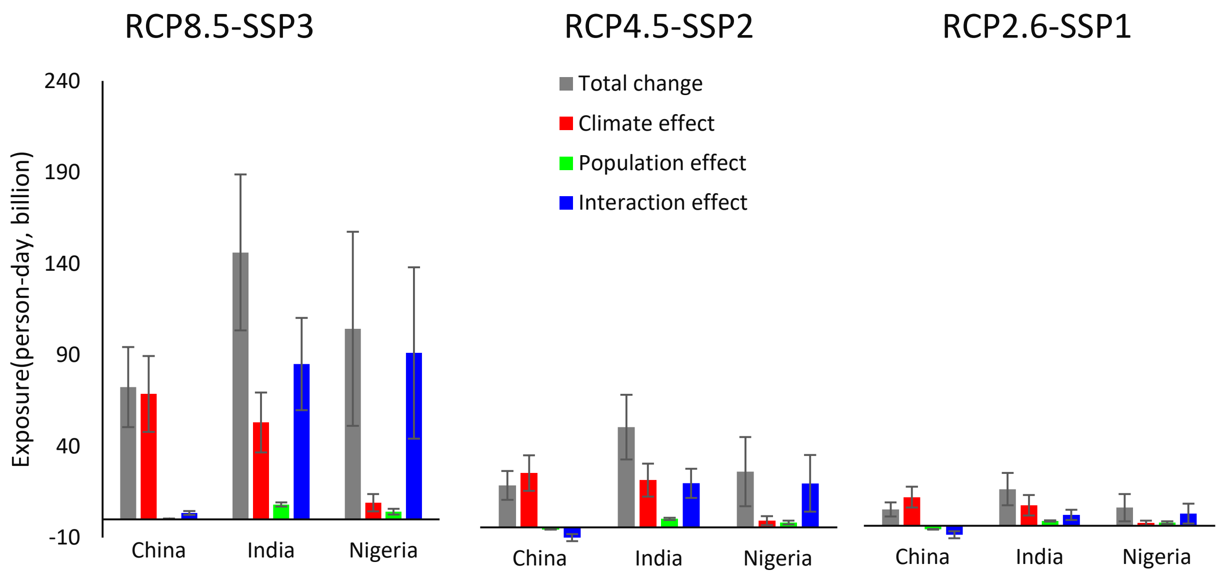

95th percentile threshold, HWD as hazard

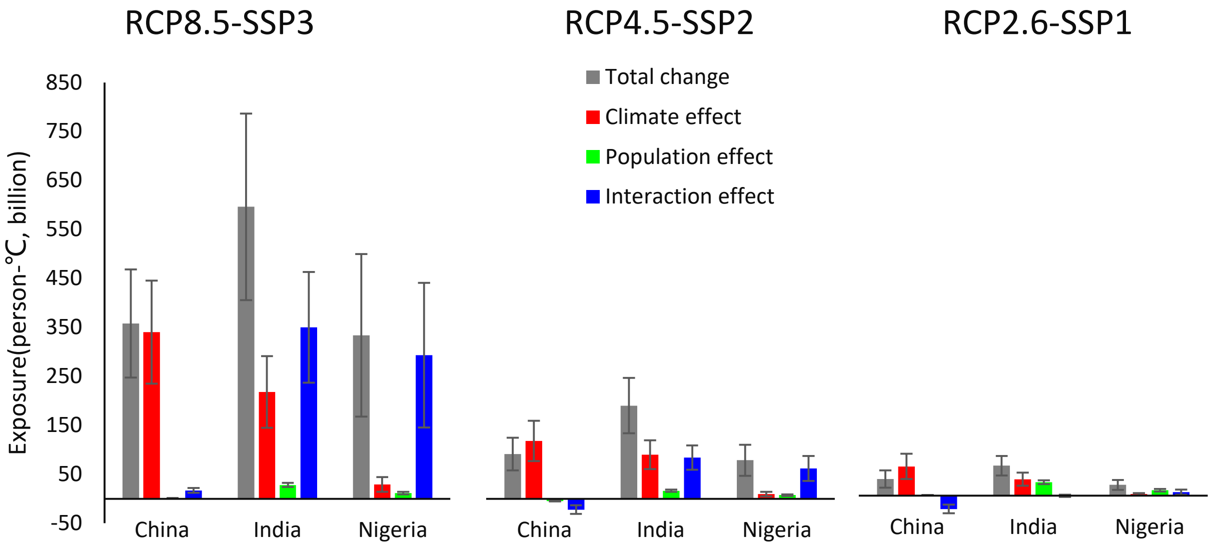

Supplementary Figure S23. Decomposition of aggregate country-level projected change in exposure for China, India, and Nigeria under three emissions scenarios. Multi-model average increase in total projected exposure change (gray), exposure change from the climate effect (red) keeping population constant; exposure change from the population effect (green) keeping climate constant; and exposure change from the interaction effect (blue) between climate and population – see Methods Section for details. Error bars illustrate the standard deviation in total projected exposure change across the models for each country/effect. First row is the results with 95th percentile HWD as hazard; second row is used 97.5th percentile HWD as hazard; third row is the results with 95th percentile AH as hazard.

95th percentile threshold, HWD as hazard

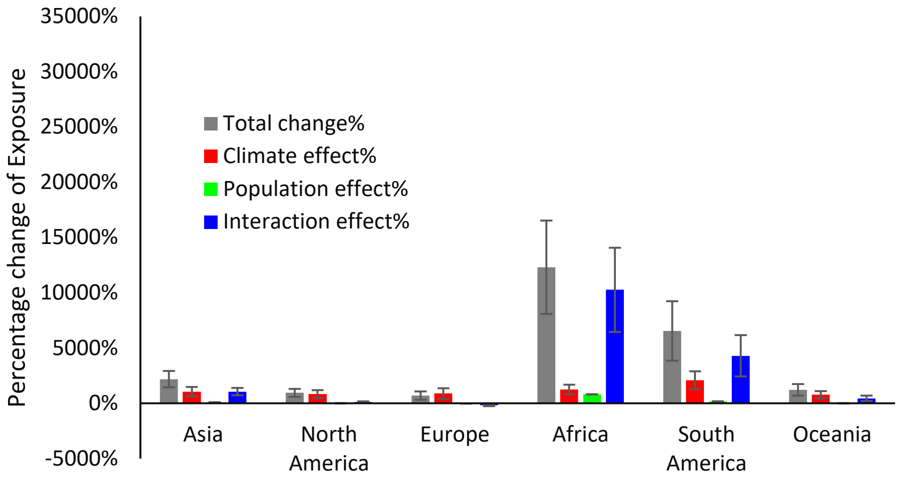

97.5th percentile threshold, HWD as hazard

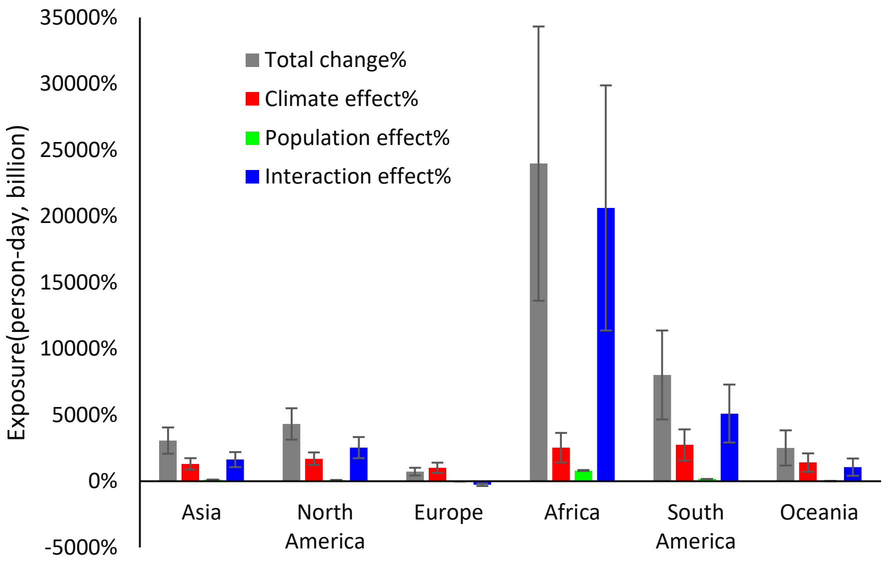

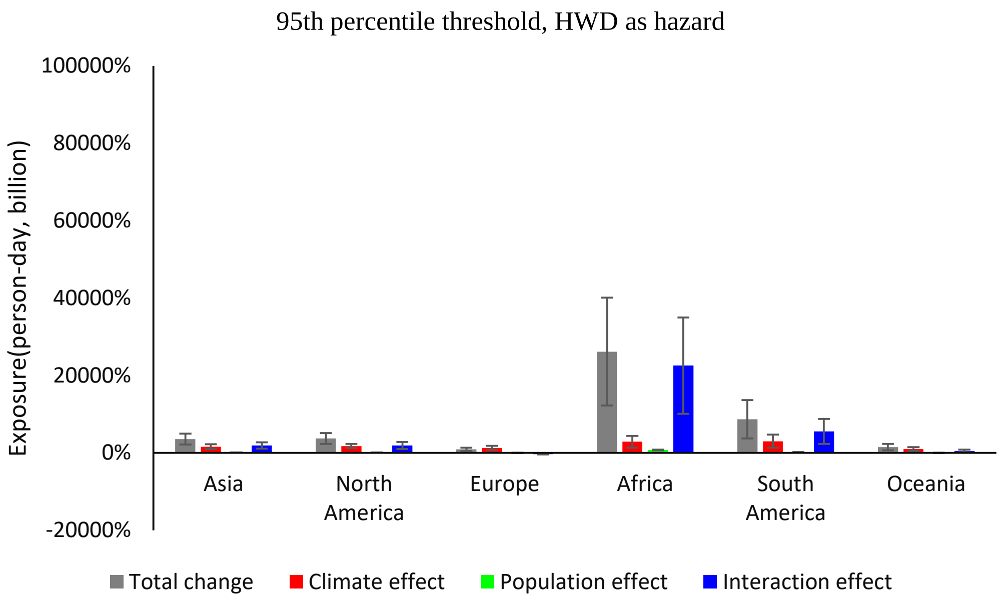

Supplementary Figure S24. Percentage increase in aggregate regional exposure under RCP8.5-SSP3. Multi-model average increase in total exposure change (gray), exposure change from the climate effect (red) keeping population constant; exposure change from the population effect (green) keeping climate constant; and exposure change from the interaction effect (blue) between climate and population – see Methods Section for details. Error bars illustrate the standard deviation in total projected exposure change across the models for each region/effect. First row is the results with 95th percentile HWD as hazard; second row is used 97.5th percentile HWD as hazard; third row is the results with 95th percentile AH as hazard.

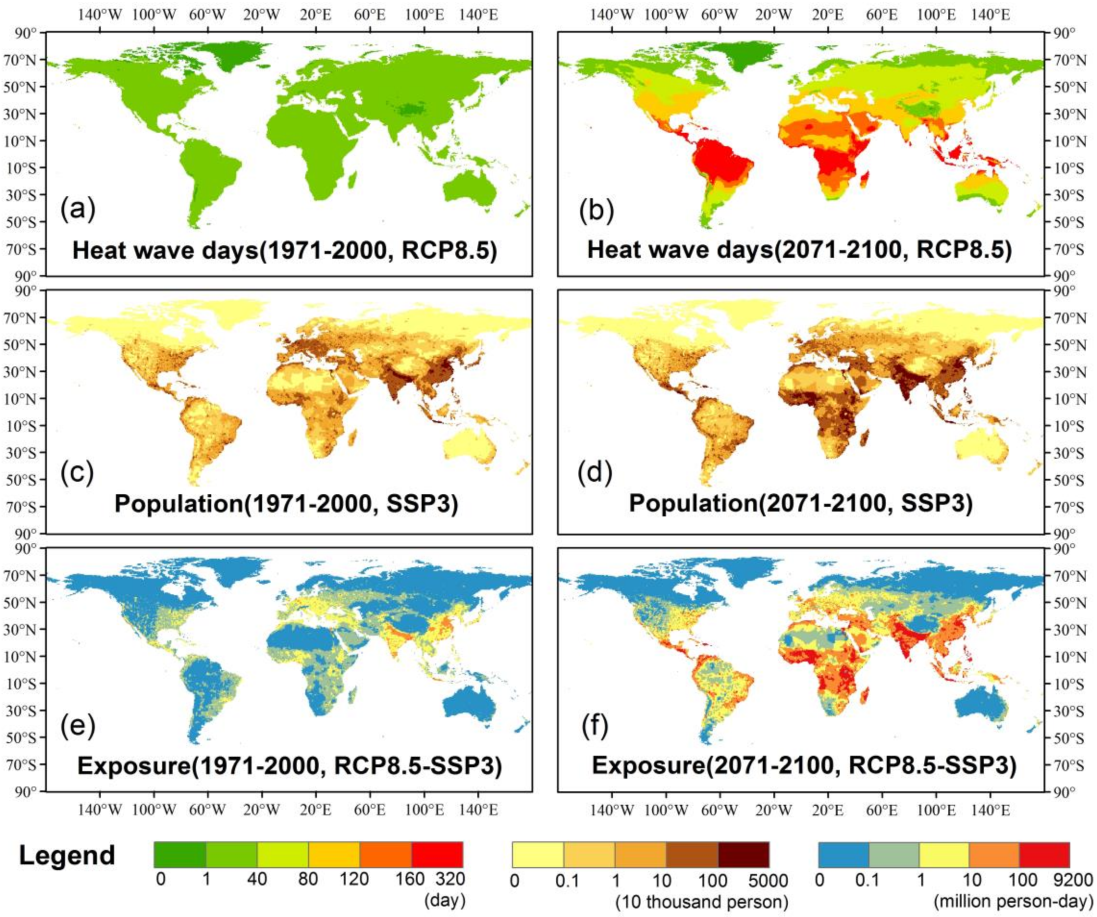

Supplementary Figure S25 Multi-model average of heat wave days (a, b; left legend; 90P6D as threshold), population (c, d; middle legend), and exposure (e, f; right legend) averaged for the period 1971-2000 (left: a, c, e) and the 2071-2100 period (right: b, d, f) under scenario RCP8.5-SSP3. These maps were generated using ArcMap 10.3, visit http://desktop.arcgis.com/en/ for more details.
